# Deep learning in neuroimaging data analysis: Applications, challenges, and solutions

**DOI:** 10.3389/fnimg.2022.981642

**Published:** 2022-10-26

**Authors:** Lev Kiar Avberšek, Grega Repovš

**Affiliations:** Department of Psychology, Faculty of Arts, University of Ljubljana, Ljubljana, Slovenia

**Keywords:** artificial intelligence, machine learning, deep learning, neuroimaging, neuroscience, data analysis, computational models

## Abstract

Methods for the analysis of neuroimaging data have advanced significantly since the beginning of neuroscience as a scientific discipline. Today, sophisticated statistical procedures allow us to examine complex multivariate patterns, however most of them are still constrained by assuming inherent linearity of neural processes. Here, we discuss a group of machine learning methods, called deep learning, which have drawn much attention in and outside the field of neuroscience in recent years and hold the potential to surpass the mentioned limitations. Firstly, we describe and explain the essential concepts in deep learning: the structure and the computational operations that allow deep models to learn. After that, we move to the most common applications of deep learning in neuroimaging data analysis: prediction of outcome, interpretation of internal representations, generation of synthetic data and segmentation. In the next section we present issues that deep learning poses, which concerns multidimensionality and multimodality of data, overfitting and computational cost, and propose possible solutions. Lastly, we discuss the current reach of DL usage in all the common applications in neuroimaging data analysis, where we consider the promise of multimodality, capability of processing raw data, and advanced visualization strategies. We identify research gaps, such as focusing on a limited number of criterion variables and the lack of a well-defined strategy for choosing architecture and hyperparameters. Furthermore, we talk about the possibility of conducting research with constructs that have been ignored so far or/and moving toward frameworks, such as RDoC, the potential of transfer learning and generation of synthetic data.

## 1. Introduction

**Figure 1:**
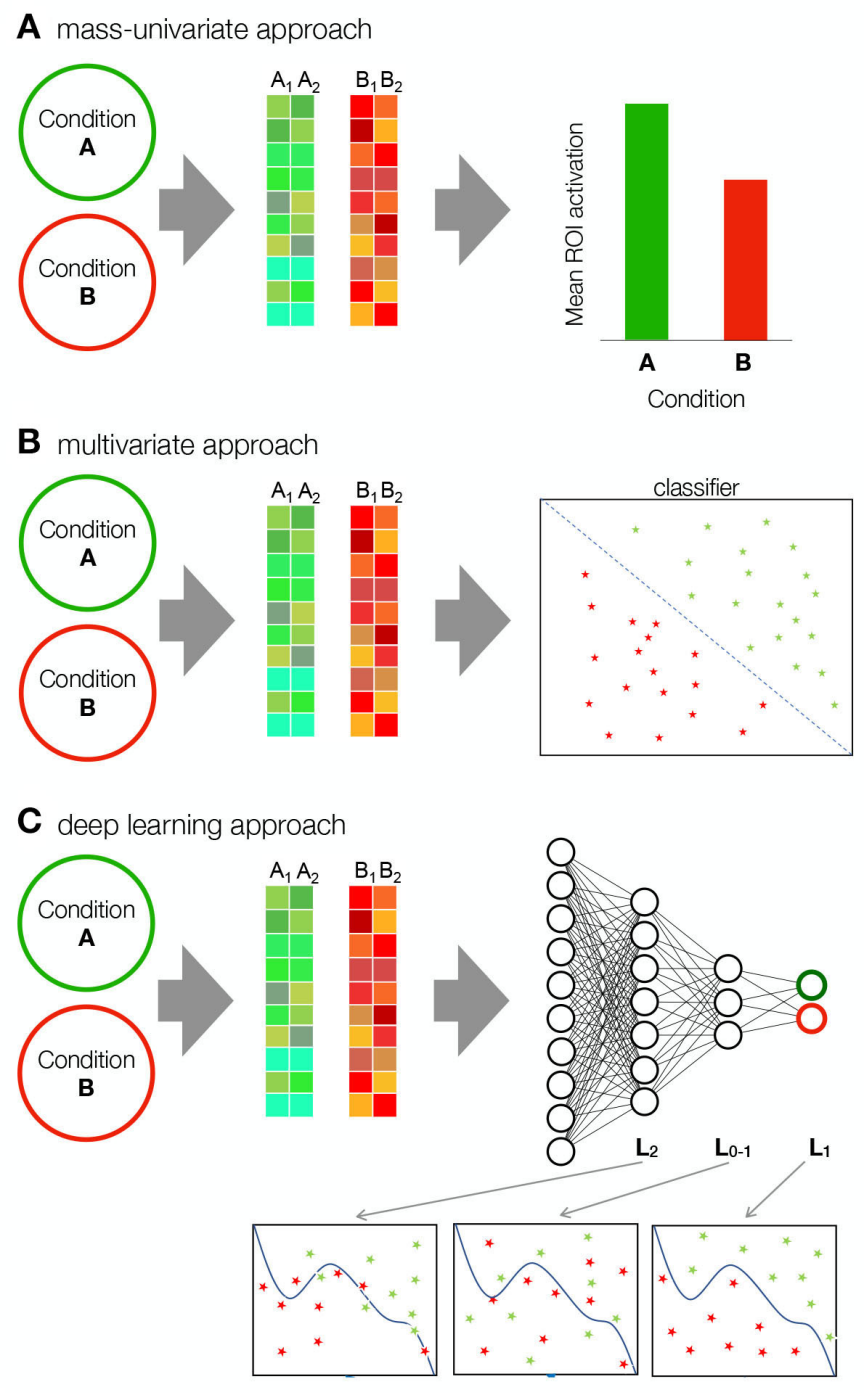
Comparison of mass univariate, multivariate and deep learning approaches. **(A)** In the mass-univariate approach, a statistic is computed independently for each sample (e.g., voxel) and compared between conditions. To assess the statistical significance of the difference, neighboring samples are usually grouped into clusters or regions of interest (ROI) over which the statistics are averaged. For example, the figure shows two samples—the activation of a group of voxels from one participant—for condition A and B. The samples in condition A and condition B are averaged and the mean activations are then compared. **(B)** MVPA methods take into account the multivariate nature of neuroimaging data. A classifier, such as SVM, is applied to the data to find a discriminant function. SVM maps the training samples to points in space to maximize the width of the gap between the two categories. **(C)** Deep learning includes multiple computational layers in form of neural networks, allowing it to form complex (non-linear) intermediary representations before classification. *L*_2_ is the second layer, while *L*_*O*−1_ and *L*_*O*_ are the output layer and its immediate precursor. The representations enabled by each layer get more complex with depth.

Imaging of the human's most complex organ—the brain—has a long past, but a short history; an attempt to paraphrase a famous psychologist (Ebbinghaus, 1908), which holds some truth. No doubt curious minds have wondered about the interior of the skull, but it is only the technological advances of the twentieth century that have allowed us to study the anatomy and function of our brains in more detail. The origins of neuroscientific research of brain function can be traced back to the 1920s, when the first electrophysiological methods were developed. However, it was not until the second half of the century that methods became available to study the structure and function of the human brain in detail. The development of functional magnetic resonance imaging (fMRI) launched neuroimaging, which has evolved into a highly complex, rigorous, and heterogeneous discipline to date (Kuntzelman et al., 2021). The beginnings of neuroimaging were marked mostly by (mass)univariate methods for data analysis (Figure 1A). The univariate approach was important for the discovery of many neural correlates and has a solid foundation (Sui et al., 2020). However, it has several limitations. Univariate methods are best suited for group-level inferences and are poorly suited for making statistical inferences at the individual level (Vieira et al., 2017). Because the univariate approach typically relies on averaging neuroimaging data across groups of participants, its generalizability is questionable. Averaging over highly heterogeneous data can lead to inaccurate and misleading results (Sui et al., 2020). Moreover, the univariate approach assumes independence of different brain regions (Vieira et al., 2017), which dilutes the information available in neuroimaging data, as we know that the functions of brain regions are highly interdependent. Later, the rapid development of neuroimaging brought about more sophisticated analysis tools that take into account the multivariate nature of neuroimaging data. Multivariate pattern analysis (MVPA) includes several methods that analyse relationships between groups of predictors (e.g., voxels) and criterion variables (e.g., behavior or cognitive state) (Figure 1B). Undoubtedly, MVPA methods represent an advance over simple univariate models. Although MVPA can include kernel operations that are sensitive to non-linear relationships in data (Treder, 2020), they are still mostly based on simple linear mathematical operations (e.g., correlation, logistic regression, support vector machines—SVM) (Kuntzelman et al., 2021). Therefore, they are unable to capture more complex patterns in neuroimaging data. This could be an important limitation given the importance of non-linear processes in the nervous system. In this review, we will look at Deep Learning (DL). DL encompasses a group of methods that use multilayer neural networks to enable representations of the underlying features of the input at different levels of complexity (Figure 1C). This computational architecture offers exciting potential for overcoming the aforementioned limitations and has therefore gained popularity in recent years in many fields, including neuroimaging.

This review is structured as follows: In the first part, we explain the basic concepts of DL, without diving too deep into mathematical details and technical implementation. In the second part, we review the most common applications of DL in neuroimaging analysis. In the third part, we discuss various challenges and possible solutions. Finally, we discuss drawbacks and future prospects of the applications of DL in neuroimaging.

## 2. Deep learning

Deep Learning (DL) is a set of representation learning methods that allows computational models composed of multiple processing layers to learn representations of data with multiple levels of abstraction. Each of these layers performs non-linear transformations of data before passing it on to another layer. In this way, very complex functions can be learned. The abstractness of the layers' representations increases with depth, amplifying aspects that are important for discrimination and suppressing irrelevant aspects. A key advantage of DL is that feature layers do not need to be hand-crafted by domain-expert engineers, but are learned through a process called back-propagation (LeCun et al., 2015).

There are numerous variants of DL models that differ in their structure, purpose, and the data they can handle. The most basic distinction is between supervised and unsupervised DL models. Both types of DL models belong to representation learning methods. The main point of difference is the usage of labeled data. Unsupervised models aim to learn the representations of an unlabeled data set, with the goal of solving tasks such as clustering, data synthesis and dimensionality reduction. In contrast, supervised DL models deal with labeled data. Their goal is to learn the probability distribution for each label to solve regression or classification problems (LeCun et al., 2015; Goodfellow et al., 2016).

**Figure 2:**
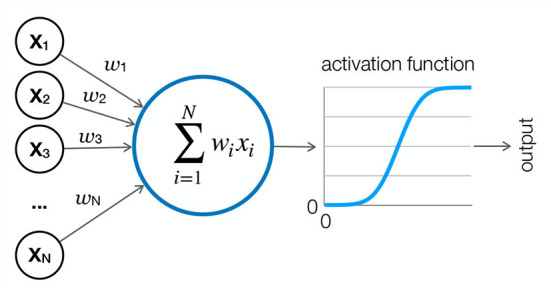
Artificial neuron. An artificial neuron is the basic computational unit of a neural network. It receives weighted inputs from neurons in the previous layer, sums them, and applies an activation function such as sigmoid or ReLu to form an output, which is usually a value between 0 and 1. The output is then either passed to the next layer or read out.

Despite their different forms, the DL models share some essential common features that are worth describing. Each DL model has a specific architecture. The architecture describes the structure of a model. The building blocks of all DL models are artificial neurons (Figure 2). Simply put, artificial neurons are basic computational units that receive inputs and convert them into outputs. Neurons are arranged hierarchically in multiple layers, with each layer receiving information from neurons in the previous layer. The connections between neurons are weighted so that the signal can either increase or decrease in proportion to its contribution to the learning task. The sum of the weighted inputs is transformed using a transformation function such as sigmoid or ReLu. When the networks contain feedback connections, they are called recurrent neural networks (RNN). Each model aims to reduce the cost or loss of its objective function. The loss is the measure of the difference between the current and desired output of the model and can be formalized using different functions, depending on the type of the problem. Two common examples of loss functions are mean squared error (MSE; Equation 1) and cross-entropy (Equation 2).

**Figure 3:**
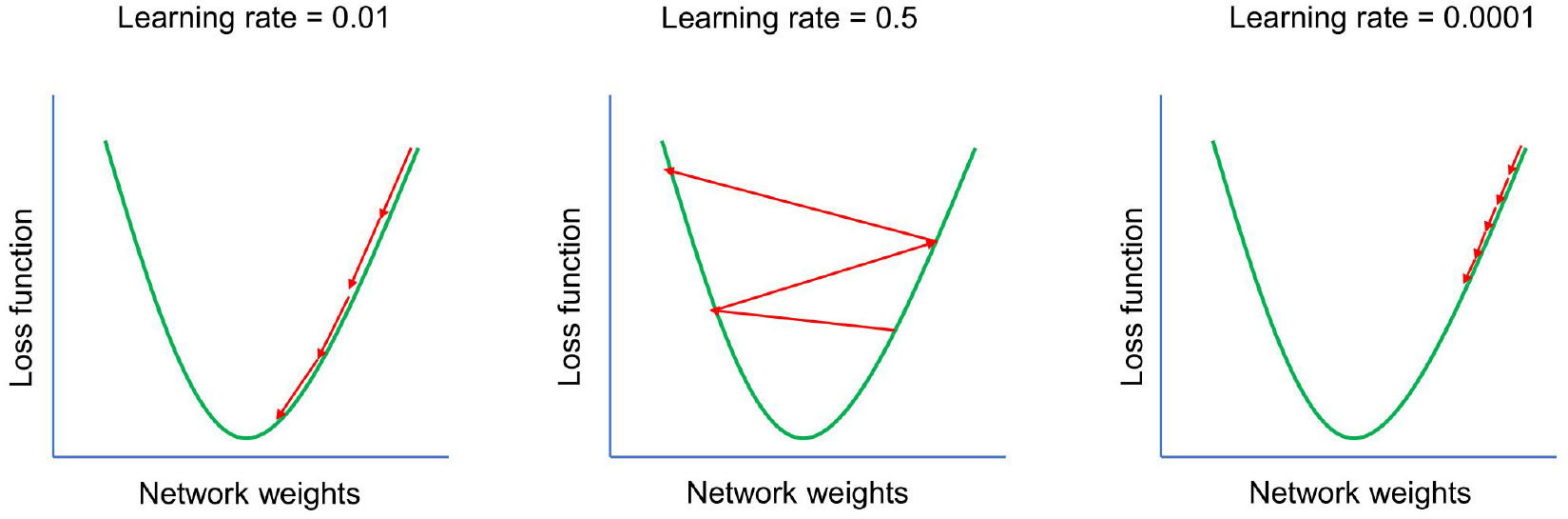
Effect of learning rate. Learning rate—one of the most important hyperparameters. If the learning rate is set too high, the model will start to diverge—the loss function of the model will increase and the accuracy of the model will decrease. If the learning rate is set too low, the model will take too long to converge. The goal is to set the learning rate to the optimal value.

During a learning phase, loss can be minimized using various learning algorithms (optimizers), of which gradient descent (Equation 3) is the most basic. The rate at which the optimizer operates is determined by the learning rate (LR—Figure 3, α in Equation 3)—a hyperparameter that determines the scale of weight change in each iteration. The information flows firstly forward and then—essentially—backward. The back-propagation procedure computes the gradient of the objective function so that the weights of the model can be optimized. After several iterations, the model—if implemented correctly—should converge to the optimum (the lowest value of the loss function). The parameters that do not change by learning are called hyperparameters. They can be divided into structural hyperparameters, which affect the design of the model, and training hyperparameters, which affect the efficiency and speed of learning. Structural hyperparameters include decisions about the number of layers and neurons in each layer. Training hyperparameters include decisions about learning rate, optimizers, etc. (Yu and Zhu, 2020).


(1) $$\begin{array}{l} J\left(\theta \right)=\frac{1}{N}\overset{N}{\underset{i=1}{\sum }}{\left(h_{\theta }{\left(x_{i}\right)-y}_{i}\right)}^{2} \end{array}$$

**Equation 1**. Mean squared error loss function. MSE is one of the most commonly used loss functions. MSE is the averaged squared difference between the model's prediction (*h*_θ_(*x*_*i*_)) and the ground truth (*y*_*i*_). θ represents the parameters that must be adjusted to minimize the loss function. MSE is most often used for regression problems.


(2) $$\begin{array}{l} H\left(O,C\right)=-\overset{M}{\underset{c=1}{\sum }}y_{o,c}\log\left(p_{o,c}\right) \end{array}$$

**Equation 2**. Cross-Entropy loss function for multiclass classification problem. The value of Cross-Entropy decreases as the probability of a given sample of belonging to the true class increases. *M* represents the number of classes, y is a binary indicator (0 or 1) if class label *c* is the correct classification for observation *o*, *p* is the predicted probability that observation *o* belongs to class *c*.


(3) $$\begin{array}{l} \theta_{j}=\theta_{j}-\alpha \frac{\partial }{{\partial \theta }_{j}}J\left(\theta \right) \end{array}$$

**Equation 3**. Gradient descent algorithm. Gradient descent updates the parameters of the model (θ) with the derivative ($\frac{\partial }{{\partial \theta }_{j}}$) of the loss function *J*(θ_0_, θ_1_) scaled by the learning rate (α). This is repeated until convergence is achieved. That is, until the loss function stops decreasing.

Due to its ability to form abstract representations based on raw data and its success in image and speech recognition, DL has found its way into biomedical sciences (Brosch and Tam, 2013; Mamoshina et al., 2016; Li et al., 2017; Vieira et al., 2017). We can see a tremendous expansion of studies in the field of neuroimaging, where DL has numerous applications and far-reaching implications. Recently researchers have utilized DL for tasks such as segmentation (e.g., Zhao, 2019; Billot et al., 2020; Brown et al., 2020; Li et al., 2021; Henschel et al., 2022; Mojiri Forooshani et al., 2022; Ushizima et al., 2022) prediction of neurologic disease (e.g., Payan and Montana, 2015; Liu et al., 2017; Lu et al., 2018; Shi et al., 2018; Wang et al., 2018; Qureshi et al., 2019; Zhou et al., 2021) and psychiatric disorder (e.g., Kuang and He, 2014; Hao et al., 2015; Kim et al., 2016; Yan et al., 2017; Heinsfeld et al., 2018; Ulloa et al., 2018; Yang et al., 2021b; Loh et al., 2022; Zhao et al., 2022), trajectory of a disorder (e.g., Spasov et al., 2019; Bae et al., 2021; Dong et al., 2021; Jung et al., 2021), different tasks (e.g., Jang et al., 2017; Vu et al., 2020; Ngo et al., 2022), brain age (e.g., Levakov et al., 2020; Ren et al., 2022), personality (e.g., Bhardwaj et al., 2021), search for biomarkers (e.g., Yang et al., 2021b), motor imagery decoding (e.g., Xu et al., 2020; Dehghani et al., 2021; Fan et al., 2021), modeling different functions of the neural system (e.g., Hebling Vieira et al., 2021) and generation of synthetic data (e.g., Kazuhiro et al., 2018; Zhao, 2019; Islam and Zhang, 2020; Li et al., 2020b; Barile et al., 2021; Hirte et al., 2021; Kossen et al., 2021). DL has been applied to data of different modalities, such as structural (sMRI—e.g., Brosch and Tam, 2013; Wang et al., 2018; Vyas et al., 2022) and functional magnetic resonance imaging (fMRI—e.g., Hao et al., 2015; Kim et al., 2016; Dakka et al., 2017; Guo et al., 2017; Zeng et al., 2018), electroencephalography (EEG) (e.g., Xu et al., 2020; Dehghani et al., 2021; Fan et al., 2021; Thanjavur et al., 2021), positron emission tomography (PET) (e.g., Zhou et al., 2021; Ushizima et al., 2022), clinical measures (e.g., Zhou et al., 2021), demographic (e.g., Liu et al., 2017; Spasov et al., 2019), and genetic data (e.g., Zhou et al., 2019; Chen et al., 2021). Before we discuss DL applications in neuroimaging in greater depth, we will briefly examine some of the most common DL model architectures.

### 2.1. Convolutional neural network—CNN

**Figure 4:**
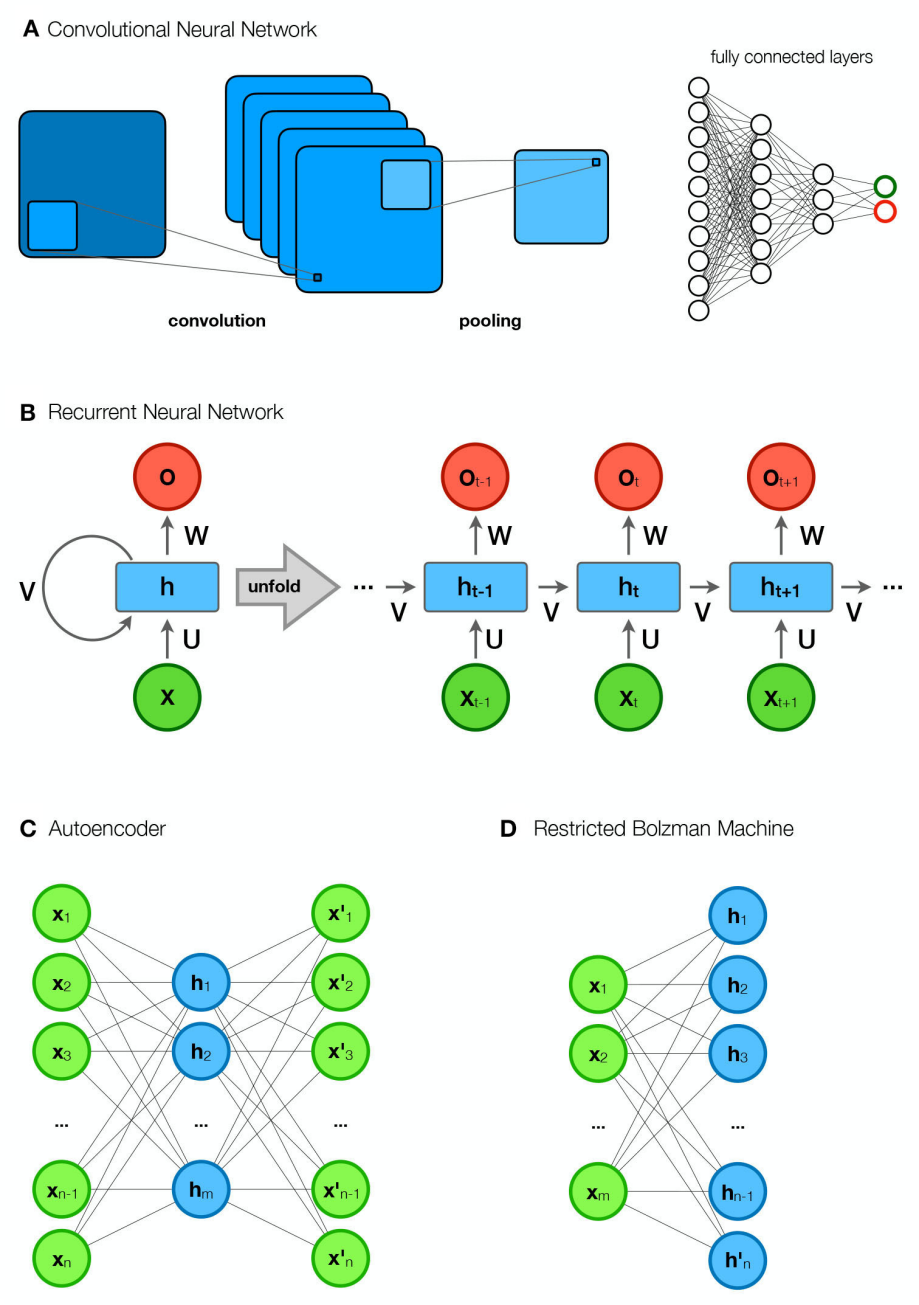
Different network types. **(A)** Convolutional neural network (CNN). The first part of a CNN consists of convolutional layers that perform convolutions to form feature maps. The feature maps of each layer are connected to the local patches in the feature maps of the previous convolutional layer through a matrix of weights called filter banks. A convolutional layer is usually followed by a pooling layer that reduces the size of the image. The “convolutional part” is followed by fully connected layers that form representations and perform classification. **(B)** Recurrent neural network (RNN). The figure shows an rolled up (compressed) and a unrolled diagram of an RNN. In the unrolled diagram, we see multiple time steps (layers). An element in a sequence (e.g., a word in a sentence) belongs to each of the layers, while the elements from the previous layers are stored in hidden states (*h*). Predictions (*y*), such as the next word in a sentence, are based on the input (*x*) and the hidden states (*h*). **(C)** Autoencoder (AE). AE encodes the input data (*x*) into its approximation within the hidden representation (*h*) and reconstructs it into new outputs (*x*′). **(D)** Restricted Bolzmann Machine (RBM). The RBM consists of visible units (*x*) and hidden units (*h*). Its goal is to learn meaningful dependencies of the visible units in the hidden layer.

A convolutional neural network (CNN) (Figure 4A) is a specific subtype of a deep neural network (DNN) that applies a mathematical operation called convolution in at least one of its layers. Roughly speaking, a convolution is an operation on two functions that produces a third function. To illustrate this, we will use the example described by Goodfellow et al. (2016). Suppose we want to measure the location *x* of a moving object at different times *t*. We can describe this with a simple function *x*(*t*). However, our measurement is subject to error. Therefore, we want to average several measurements to get a more accurate result. Since more recent measurements give a better estimate of the current location, we want to use weighted averages with respect to the age of the measurement *w*(*a*). The operation of applying *w*(*a*) to *x*(*t*), by which we obtain a new function *s*(*t*) = (x*w)(*t*)—the smoothed estimate of the location of the moving object—is called convolution. The first argument (*x*) is called the input, while the second argument (*w*) is called the kernel.

The input to a convolution layer is usually a multidimensional array of data, such as a 2D image. The kernel, is also a multidimensional array, with non-zero values at specific locations learned by the learning algorithm. The output of a convolution is a feature map (Goodfellow et al., 2016). The units in each convolutional layer are organized into feature maps that are connected to local patches in the feature maps of the previous convolutional layer *via* a matrix of weights called a filter bank. All units in the same feature map are assigned the same filter bank. Units in other feature maps share other filter banks (LeCun et al., 2015). This type of architecture allows the model to capture locally meaningful representations while making it robust to variable spatial locations of motifs. Almost all CNNs consist of a pooling layer whose task is to merge semantically similar features of the convolutional layer output. Typically, multiple stacks of convolutional layers and pooling layers are placed in front of fully linked layers. The aforementioned features allow CNNs to exploit the internal hierarchical structure of the input data (LeCun et al., 2015). Feature maps become more abstract with each convolutional layer; while lower layers capture features such as shape or direction, higher layers can learn to differentiate between semantic categories (Zeman et al., 2020).

### 2.2. Recurrent neural network—RNN

The recurrent neural network (RNN) (Figure 4B) is a subtype of the deep neural network that is capable of processing sequential data (such as speech or language). The key feature of an RNN is the sharing of parameters. Sharing parameters at different points in the model allows generalization between examples of different shapes (e.g., length) (Goodfellow et al., 2016). RNNs process each element of a sequence separately and store information about all elements in hidden units (state vectors). Each node with hidden units represents a point in time (the position of the element in the sequence) and receives information from the previous node representing the previous point in time. The dynamic system allows the model to capture the temporal dimension of the data (LeCun et al., 2015).

### 2.3. Autoencoder—AE

Two components of an autoeconder (AE) are an encoder and a decoder (Figure 4C). The encoder transforms the input data into an internal representation. The task of the decoder is to reconstruct the internal representation into output data that is an approximation of the input data. An AE is intended to be constrained in some way so that it does not learn to output an exact copy of the input data, but instead learns meaningful features of the data. This is based on the premise that high-dimensional data is concentrated around a lower-dimensional manifold. The goal of an autoencoder is to learn the structure of this manifold. The training of an AE should include an architectural constraint or regularization penalty. The specific features of the training allow the model to learn only the representations of the vectorial directions necessary to reconstruct the input data. There are several types of AEs, e.g., undercomplete AE, whose internal representation has a smaller number of dimensions than the input data, sparse AE, which adds a penalty term to the reconstruction algorithm, denoising AE, which changes the cost function so that the input data is treated as corrupted and must be repaired during reconstruction, and variational AE, whose latent vector consists of probability distributions. AEs have been successfully used in dimensionality reduction and information retrieval tasks (Goodfellow et al., 2016).

### 2.4. Restricted Bolzmann machine—RBM

A Restricted Bolzmann Machine (RBM) is a generative model consisting of two types of units: visible and hidden (Figure 4D). The visible units correspond to the input data (e.g., one unit for each pixel of an image), while the hidden units extract the meaningful dependencies of the visible units (features). RBM is set to learn a probability distribution that matches the probability distribution of the training data. RBMs can also be viewed as building blocks of Deep Belief Networks (DBNs). The idea is that each RBM receives the values of the hidden units of the previous RBM as input data. In this way, the deeper building blocks are able to learn higher level features of the data (Fischer and Igel, 2012).

### 2.5. Generative adversarial networks—GANs

**Figure 5:**
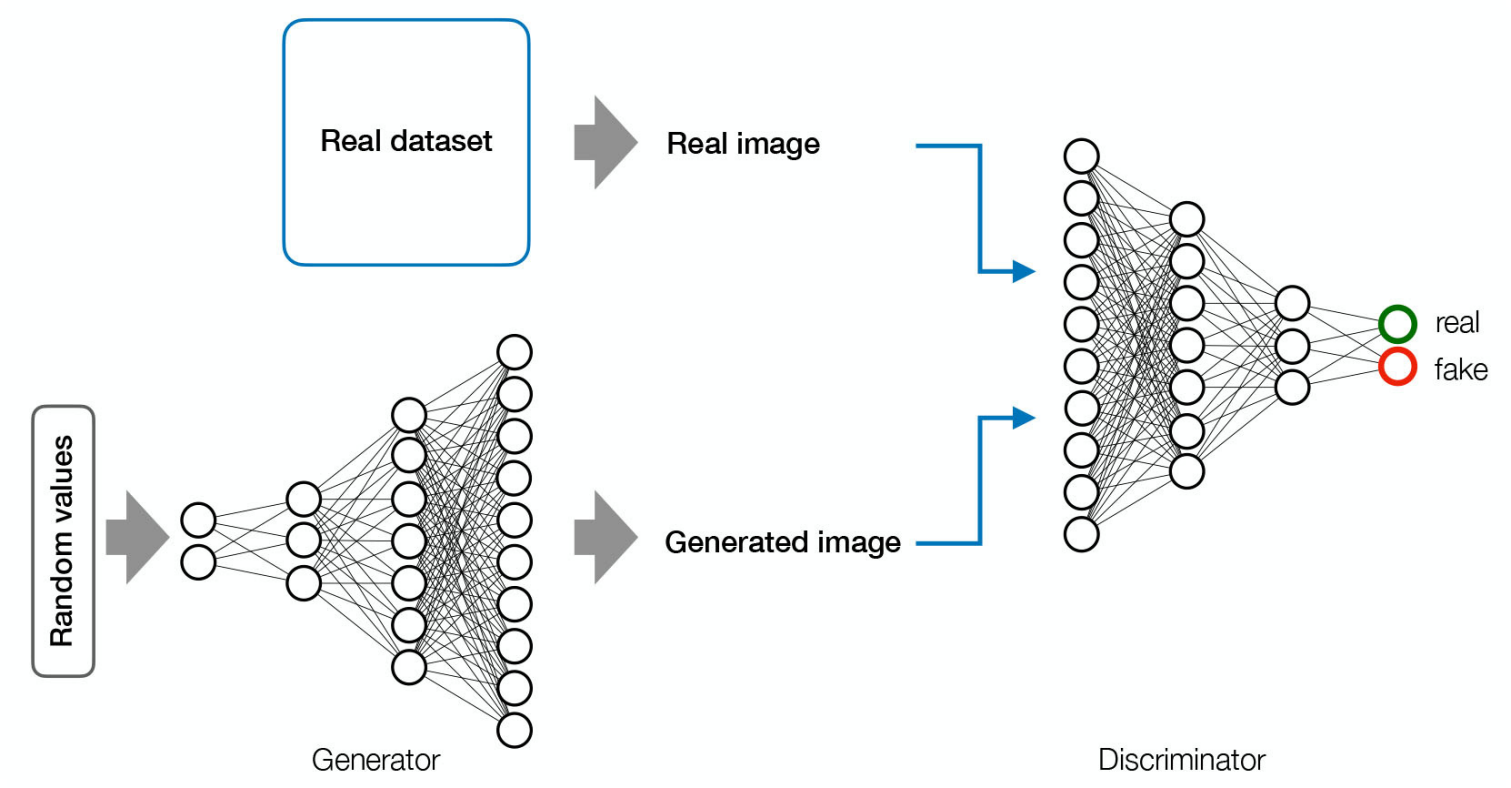
Generative Adeverserial Network—GAN. GAN Consists of two adversarial models. The generator (inverted CNN) draws random values from a uniform distribution to generate images. The generated images are sent to the discriminator (CNN) along with the images from the real data set. The goal of the discriminator is to learn to classify images as real or fake. The goal of the generator is to deceive the discriminator. The backward flow of information allows the discriminator to adjust its parameters correctly and generate more realistic images. A GAN is converged when the discriminator classifies at chance.

Generative Adversarial Networks (GANs) are generative models consisting of two adversarial components (Figure 5). The *generator* draws random values from a uniform distribution to construct images. These images are then sent to the *discriminator* along with images from a real data set. The discriminator is a CNN whose job is to distinguish between *real images* from the training data set and *fake images* generated by the generator. Through back-propagation, the generator learns to construct images with increasing degrees of deception. The ultimate goal is to reach a level of synthetic image quality where the discriminator is no longer able to classify images as real or fake above chance (Goodfellow et al., 2014). GANs have achieved impressive performance in image synthesis, but are quite difficult to train because they are highly unstable (Goodfellow et al., 2016).

## 3. Deep learning applications in neuroimaging

In this next section, we will discuss the applications of DL in neuroimaging data analysis that we have identified as the most common. Certainly other applications such as noise reduction, artifact detection, and resolution enhancement have been tried, but not as frequently. An important line of research that we also did not include is the use of DL as a computational model for cognitive functions, such as vision. This is because we want to focus on DL as a statistical procedure rather than as a computational model for brain function *per se*.

### 3.1. Prediction

Predicting future outcomes based on present data is one of the most important tasks of science and the field of statistics. Accurate prediction enables appropriate response. Statistical models of the highest quality are therefore invaluable not only for basic research but also for practical applications. The development of statistics has produced sophisticated models for univariate and multivariate prediction of categorical and continuous variables. Indeed, prediction is one of the most widely recognized goals and one of the key accomplishments of Deep Learning. Currently, the discriminative DL models, of which the two most commonly used representatives are CNNs and RNNs (Figure 4) and their architectural modifications, represent state-of-the-art models for multi-array and sequential data classification, respectively (LeCun et al., 2015).

**Figure 6:**
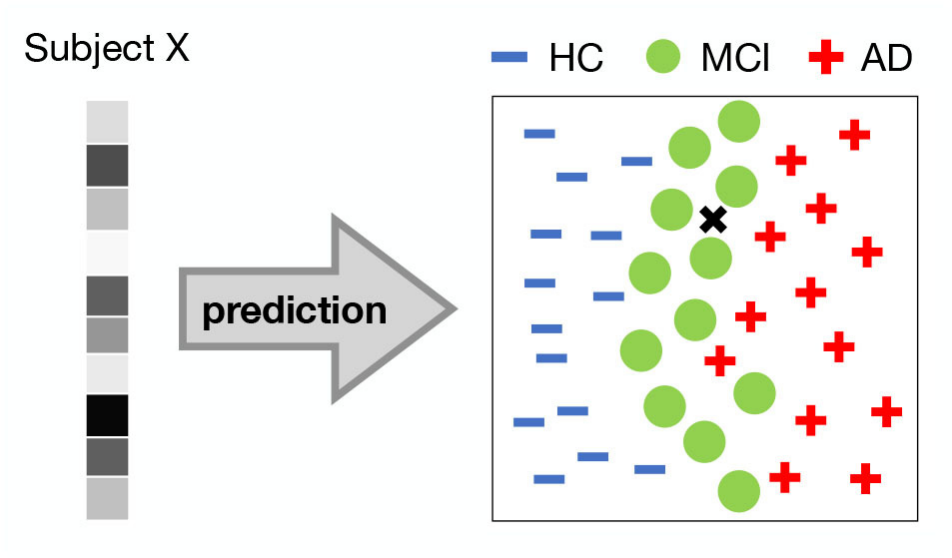
Classification task. 2-dimensional illustration of a DL representation in a 3-way classification task between healthy controls (HC), patients with Minimal Cognitive Impairment (MCI) and Alzherimer's Disease (AD). A pattern of voxel activations (e.g., from a ROI) of a person is input to a DL model. The DL model forms complex representations and classifies the subject into one of the groups. In this example, the DL model would classify the subject as suffering from MCI.

Arguably, the most important prediction is that of pathology. Logically, the pathology of brain structure and/or function that we can observe from neuroimaging data can lead to a prediction of a specific symptomatic outcome. Most studies that have applied discriminative DL models have focused on diagnostic prediction. That is, they discriminated between healthy controls and diseased individuals based on neuroimaging data (e.g., Figure 6). Although both psychiatric disorders (as defined by DSM-5) and neurological disorders can be the result of either functional or structural brain changes at different levels of observation, existing studies tend to use functional data to predict psychiatric disorders and structural data to predict neurological disorders. DL approaches using fMRI data have been used to diagnose schizophrenia (SCZ) (Plis et al., 2014; Kim et al., 2016; Dakka et al., 2017; Yan et al., 2017; Ulloa et al., 2018; Zeng et al., 2018; Chen et al., 2021; Hu et al., 2022), autism spectrum disorders (ASD) (Guo et al., 2017; Heinsfeld et al., 2018; Shao et al., 2021; Yang et al., 2021b; Kashef, 2022; Zhang et al., 2022a), attention deficit and hyperactivity disorder (ADHD) (Kuang and He, 2014; Deshpande et al., 2015; Hao et al., 2015; Zou et al., 2017; Mao et al., 2019; Zhao et al., 2022), posttraumatic stress disorder (PTSD) (Sheynin et al., 2021; Yang et al., 2021a), bipolar disorder (BD) and schizoaffective disorder (Yan et al., 2022), while sMRI data have been used to diagnose Alzheimer's disease (AD) or/and mild cognitive impairment (MCI) (Brosch and Tam, 2013; Gupta et al., 2013; Chen et al., 2015; Payan and Montana, 2015; Hosseini-Asl et al., 2018; Lu et al., 2018; Wang et al., 2018), Parkinson's disease (PD) (Shen et al., 2020; Vyas et al., 2022), Huntington's disease (HUN) (Plis et al., 2014), cerebrovascular disorders (Liu et al., 2019), and tumor (Van Hai and Amaechi, 2021).

In our literature review, similar to Vieira et al. (2017), we observed a slight tendency for better results in studies using structural data to diagnose neurological disorders than studies using functional data to diagnose psychiatric disorders. However, it should be noted that structural data sets are generally larger than functional data sets and that the neural bases of psychiatric disorders are less well-understood than that of neurological disorders. In addition, the temporal dimension of functional data makes them and the associated diagnosis of psychiatric disorders more complex. Other functional data (e.g., EEG) have been used to detect epilepsy (Thodoroff et al., 2016; Golmohammadi et al., 2019; Zhang et al., 2022b) concussion (Thanjavur et al., 2021), major depressive disorder (MDD) (Korda et al., 2021; Loh et al., 2022) and outcome of comatose patients (Jonas et al., 2019). Some studies went further and employed DL to predict disorder trajectory (Jung et al., 2021) or state of a progressive disease (Liu et al., 2014, 2015, 2017; Li et al., 2017; Helaly et al., 2021; Zhou et al., 2021) and gender differences in ASD (Supekar et al., 2022).

A number of studies addressed prediction outside the field of brain pathology. Task-based fMRI data have been used to predict task state (Jang et al., 2017; Hu et al., 2019; Vu et al., 2020; Wang et al., 2020b; Jiang et al., 2022; Ngo et al., 2022), while EEG data were used to predict attentional state (Zhang et al., 2021), sleep stage (Abou Jaoude et al., 2020; Akada et al., 2021) and brain age (Levakov et al., 2020; Niu et al., 2020; Ning et al., 2021; Ren et al., 2022), recognize emotions (Wang et al., 2020a; Ramzan and Dawn, 2021; Bagherzadeh et al., 2022; Xiao et al., 2022), detect P300 (Solon et al., 2019; Borra et al., 2021), cortical oscillatory activity (Abdul Nabi Ali et al., 2022) and cortical activity during sleep (Li et al., 2020a). Recently, several studies have used DL to decode motor imagery (Hassanpour et al., 2019; Ebrahimi et al., 2020; Xu et al., 2020; Dehghani et al., 2021; Fan et al., 2021), which is important in brain-computer interface.

The promise of DL is more than obvious. Most studies comparing DL to other ML methods (e.g., shallow and linear models) showed the superiority of DL (Kim et al., 2016; Dakka et al., 2017; Guo et al., 2017; Heinsfeld et al., 2018; Shi et al., 2018; Ulloa et al., 2018; Zeng et al., 2018; Yan et al., 2019). Combining data from multiple modalities (e.g., MRI and PET) to train the model has shown promise. Most authors report an increase in accuracy compared to unimodal training methods (Chen et al., 2015; Liu et al., 2015; Zou et al., 2017; Lu et al., 2018; Shi et al., 2018; Ulloa et al., 2018; Niu et al., 2020; Zhou et al., 2021; Ren et al., 2022). Several studies also reported better results using 3D data instead of 2D data (Payan and Montana, 2015; Vu et al., 2020; Hu et al., 2022; Vyas et al., 2022). Transfer learning also provided promising results. It worked not only when the pretraining was performed with neuroimaging data set (Payan and Montana, 2015; Heinsfeld et al., 2018; Wang et al., 2018, 2020a,b, 2021; Golmohammadi et al., 2019; Dehghani et al., 2021; Helaly et al., 2021; Yang and Hong, 2021; Bagherzadeh et al., 2022; Balboni et al., 2022; Jiang et al., 2022; Ngo et al., 2022), but higher performance was also observed when pretraining was conducted with natural images (Gupta et al., 2013).

### 3.2. Interpretation

The ultimate goal of science is not only to predict future outcomes, but also to understand what the prediction is based on. DL models are often referred to as “black boxes” because the representations they construct are highly complex and difficult for human observers to interpret. This poses a risk to all areas where artificial intelligence (AI) is used. In medicine, for example, the inability to propose a valid interpretation of a model could lead to the use of models that achieve high accuracy on the one hand but exploit clinically irrelevant features of the data for their predictions on the other (Vieira et al., 2017). In neuroscience, the ability to understand the representations of models would also facilitate the discovery of novel biomarkers and thus a better and more mechanistic understanding of a disorder or brain states (Durstewitz et al., 2019).

Fortunately, in parallel with the development of AI, the field of explainable AI (XAI) has recently emerged. XAI does not represent a single recipe for understanding AI decision-making, but is rather a conceptual framework in which many different methods are being developed with different underlying assumptions about what “explainable” means (Ras et al., 2020). Nevertheless, there are several general *traits*—evaluation criteria—for XAI. First, *confidence* is a trait that assesses the congruence of the model's underlying computations with the human observer's thought process. Namely, if an AI decision is based on the same aspects of the data as the human observer's decision, then confidence of the model is high. Second, *trust* is a criterion based on the model's performance on various metrics, most commonly test accuracy. If a model is highly accurate, then it can be trusted. Third, *safety*, a multi-faceted category that is mainly concerned with the reliability of the model under various working conditions. Finally, *ethics*, the most elusive of all evaluation criteria. The cultural relativity of ethics prevents us from imprinting a universal moral code on a model. The ability to understand whether an AI's decision is consistent with the moral code of the environment in which it operates is therefore a more viable solution (Ras et al., 2020).

All of the aforementioned traits are important to the field of neuroimaging. The first two (confidence and trust) are specifically valuable for research purposes, while the last two (safety and ethics) are invaluable for practical (clinical) applications. Since this review focuses mainly on the impact of DL on basic research, we will look in more detail at methods related to the first two criteria. We should emphasize that confidence should be understood in a particular way. Neuroimaging data are very complex and difficult for human observers to understand. Therefore, the sole goal should not be that the underlying computations on the basis of which a model makes predictions resemble those of a human observer. Rather, these latent representations should facilitate new ways of understanding the data.

According to Ras et al. (2020) XAIs can be divided into three groups: Visualization methods, Distillation methods, and Implicit methods. Visualization methods attempt to highlight the aspects of the input data that contribute highly to the output. Distillation methods are performed after training and typically involve encoding the learned knowledge into a “white-box” system suitable for human interpretation. Intrinsic methods involve models that provide explanations as part of their output.

By far the most common approach in neuroimaging is visualization. Visualization methods can be divided into backpropagation-based methods and perturbation methods. The former are based on the evaluation of gradient signals sent from output to input, while the latter deform the input and examine the performance of the model after the change. As reported by Vieira et al. (2017), deconvolutional methods, which belong to the backpropagation group, are the most commonly used. In the original paper (Zeiler and Fergus, 2013), deconvolutional method is defined as an inverted operation of a CNN. Convolutional operations are replaced by deconvolutional operations, while pooling is replaced by unpooling. Consequently, the data flow from a given neuron to the input image is inverted. In this way, one can check which part of the image contributes most to the activation of the neuron.

**Figure 7:**
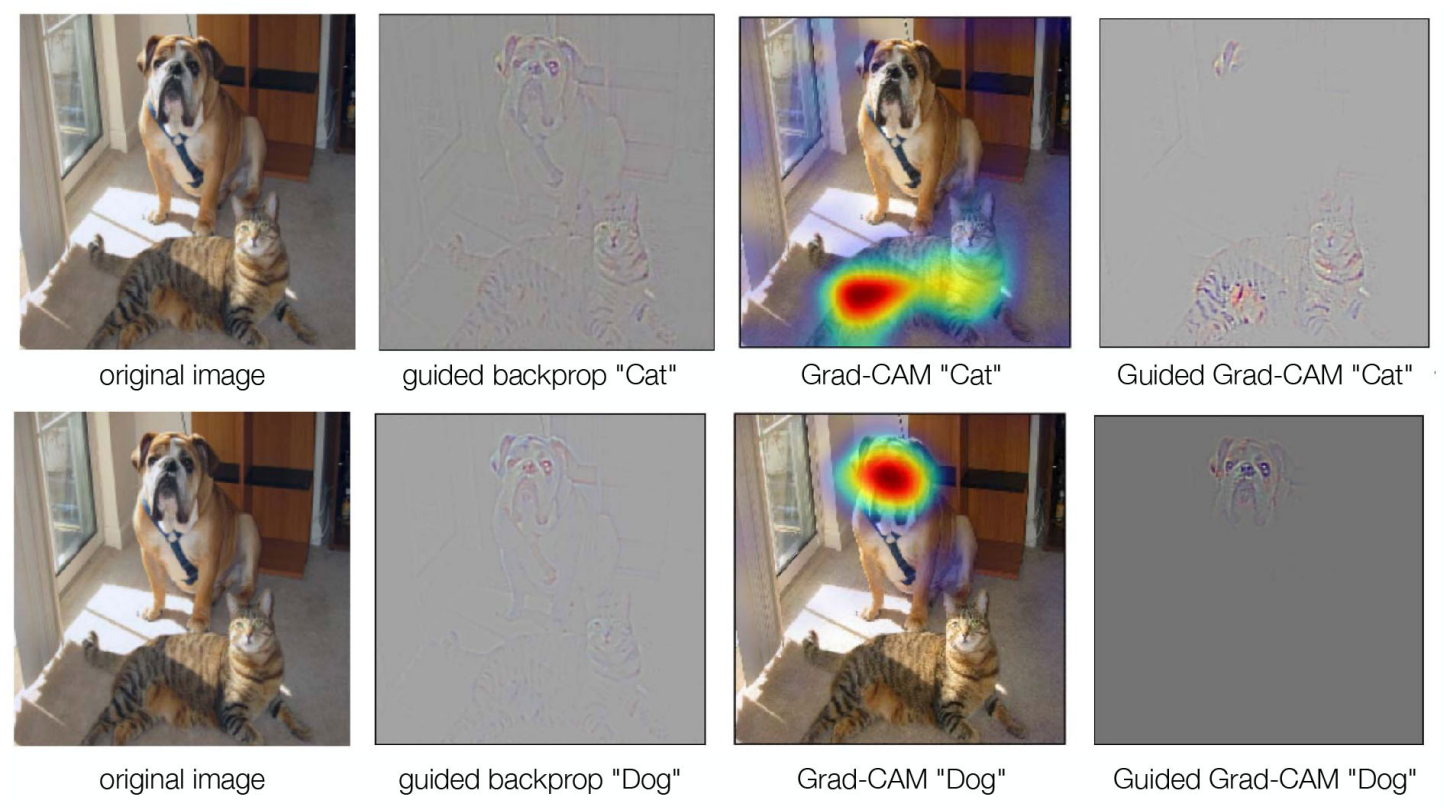
GBP, Grad-CAM, and Guided Grad-CAM. GBP is identical to a backward pass, but only considers the highest gradient when passing through a non-linearity. GBP is high-resolution, but not class-discriminating. Grad-CAM, on the other hand, requires a forward pass to obtain a raw score for an image. The gradient of the target class is set to 1, while all other gradients are set to 0. Then a backward pass through the feature maps of interest is performed. This results in coarse localizations that contribute the most to the classification. To obtain high-resolution, class-discriminative results, we can combine GBP and Grad-CAM to Guided Grad-CAM (Selvaraju et al., 2020).

Guided back-propagation (GBP) (Figure 7) is an extension of the deconvolutional method. While deconvolutional methods require a forward pass of an image before obtaining a discriminative reconstruction, GBP does not. Thus, it is not conditioned on a single image, which means that it can learn latent features directly. In essence, GBP is identical to a backward pass, except that it only considers the top gradient when passing a non-linearity. It can therefore provide more accurate results, especially for the higher layer (Springenberg et al., 2014).

While GBP provides high-resolution results, it is not class discriminating. That is, the output image is not exclusively focused on the target concept. One solution to this problem is Gradient-weighted Class Activation Mapping (Grad-CAM Selvaraju et al., 2020) (Figure 7). In Grad-CAM, a forward pass of an image is first performed to obtain a raw score. The gradient of the target class is set to 1, while all other gradients are set to 0. A backward pass through the feature maps of interest is then performed. This results in coarse localizations that contribute most to the classification. The method can be combined with GBP to obtain high-resolution results (Selvaraju et al., 2020) (Figure 7).

Yet another alternative is Layer-wise Relevance Propagation (LRP Bach et al., 2015), a method that measures relevance instead of sensitivity. That is, the strength of the connection input or pixel to a given network output (Ras et al., 2020).

The listed methods have all been used in neuroimaging analysis. For example, GBP was used by Wang et al. (2020b) to study the features learned from 3DCNN, whose task was to classify task states based on fMRI data. Grad-CAM was used in studies where CNNs were used to examine features learned for AD prediction based on PET data (Ushizima et al., 2022), brain age prediction based on sMRI and blood parameters (Ren et al., 2022), emotion recognition based on electrode frequency distribution maps (Wang et al., 2020a), and comatose patient outcome based on EEG (Jonas et al., 2019). LRP was used in a study in which the authors trained a DNN to predict SCZ based on resting-state functional connectivity MRI data (Yan et al., 2017).

Several studies also used a simpler version of visualization that did not directly measure the contribution of input data aspects. These studies typically used “linear projection”, a technique in which a feature (feature vector) in a layer is defined as a linear combination of units from a previous layer that are connected to it. In other words, a feature vector is a product of features from a lower layer and a matrix of weights connecting the lower layer features to the higher layer. A simple forward pass can be applied to extract the features of interest. Visualization of these features is then followed by dimensionality reduction techniques such as PCA or t-SNE (van der Maaten and Hinton, 2008) or techniques such as representational similarity analysis (RSA Kriegeskorte, 2008) and software toolboxes such as BrainNet Viewer (Xia et al., 2013) and circularGraph (Kassebaum, 2022).

While few studies have used perturbation methods (e.g., Ushizima et al., 2022), there are numerous studies that use other forms of quantitative techniques. Many studies used perturbation methods, in which the input (e.g., a brain area or functional network) is modified and the changes in the output (e.g., prediction accuracy) are examined (Vieira et al., 2017). The leave-one-out technique (LOO), which measures a target matrix (usually accuracy) after omitting a feature, is very popular (e.g., Yan et al., 2019, 2022; Niu et al., 2020). The basic idea of LOO is that the importance of a feature is proportional to the decrease in accuracy after its omission. Other quantitative methods include measuring the ability of a layer or feature to discriminate between groups with Fisher's Z-scores and t-scores (Kim et al., 2016; Guo et al., 2017), F-scores (Zhang et al., 2022a), and feature stability (Liu et al., 2014, 2015).

Apart from methods discussed, some researchers have also experimented with intrinsic methods. In Jiang et al. (2022), an attentional module parallel to feature extraction was added to a 4DCNN model aimed at predicting task state based on fMRI data. Its function was to enhance discriminative representations of objects with maxpool and resblock layers. In Fan et al. (2021), the attention module was used to learn the attentional weights of EEG channels, time points, and feature maps that contribute to the decoding of motor imagery by a DNN called QNet.

An alternative to training discriminative DL models (such as CNNs and RNNs), extracting their features, and visualizing them with dimensionality reduction is to use DL models that are themselves capable of dimensionality reduction, such as AEs, RBMs, and DBNs (Figure 4), and inspecting the features they learn (e.g., Plis et al., 2014; Han et al., 2015; Guo et al., 2017; Jang et al., 2017; Heinsfeld et al., 2018).

### 3.3. Generation

One of the main problems, not only in neuroimaging but in medical imaging in general, is the scarcity of labeled data on which supervised ML algorithms can learn (Lan et al., 2020). Indeed, the abundance of data is one of the cornerstones for the success of supervised DL algorithms in areas such as computer vision. It is also a driving force in preventing overfitting. While state-of-the-art models in computer vision have been trained on data sets with hundreds of thousands to millions of images (e.g., Imagenet with more than 14 million images; Deng et al., 2009), data sets for neuroimaging typically include between a few hundred to thousand participant (e.g., ADNI with 819 participants; Petersen et al., 2010). Projects are underway to collect neuroimaging data from multiple sites into a single data set (e.g., HCP; Van Essen et al., 2013), but massive data sets optimal for training a model with several million parameters are not expected in the near future.

One way to circumvent this serious limitation is to create artificial (synthetic) data. A simple solution is data augmentation, i.e., modifying the existing data by various operations such as rotating, shearing, cropping, etc. Data augmentation has been applied before in the field of neuroimaging (Billot et al., 2020; He et al., 2021), but the scope of this simple method is limited because the distribution of the generated images is very similar to the existing images (Lan et al., 2020). This makes the method ill-suited for applications where the entire spectrum of a medical phenomenon (e.g., the spectrum of a psychiatric disorder) is to be examined. A more appropriate method would be able to represent the entire distribution. Indeed, such methods have been developed in the field of DL. One of the most popular methods in recent years are GANs. Given their incredible success in generating images, they have also entered other fields such as biomedical sciences (Lan et al., 2020).

GANs are considered to be able to generate images that share all essential features with images of real patients (Wolterink et al., 2021). Several authors reported encouraging results. Kazuhiro et al. (2018) tested the authenticity of “fake images” on two radiologists who showed a 44% and 71% ability to distinguish them from real images. Li et al. (2020a) reported improvement in brain tumor segmentation after using synthetic images. Islam and Zhang (2020) reported very high similarity to real images. Barile et al. (2021) demonstrated an application of GANs to structural connectivity in multiple sclerosis patients. Quantitative and qualitative analyses showed no significant differences from real images, while predictive accuracy (F1 score) increased from 66 to 81%. Hirte et al. (2021) evaluated the ability of GAN and AE to generate synthetic MRI images. Both models generated data that were very similar to the originals and exhibited a high degree of sharpness and diversity. However, the models were also found to produce groups of images that were nearly identical. Two neuroradiologists classified the vast majority (above 80%) of the generated images as genuine. Kossen et al. (2021) used GANs to generate 3DTOF MRA images for blood vessel segmentation. The results showed that their mixed-precision GAN model was able to generate images that were nearly identical to real images while reducing computational costs. Segmentation on synthetic images was close to the success level achieved by state-of-the-art models trained on real images. Another promising approach was implemented by Zhao (2019), who constructed a Bayesian conditional GAN. The model was not only able to generate highly accurate images of brain tumors, but also to propose uncertainty maps that can help practitioners decide whether to trust an image.

GANs have not only been used to generate images obtained by various imaging modalities (CT, MRI, microscopy, etc.), they are also capable of translating between imaging modalities. A subtype of GAN, called cyclic GAN, is able to map input images to different modalities. In practice, this means that images of one modality can be used to generate images of another modality (Lan et al., 2020). In neuroimaging, GANs have been most commonly applied to CT, MRI, and PET (Laino et al., 2022).

### 3.4. Segmentation

Image segmentation—a process of dividing an image into semantically meaningful homogeneous subunits—is an important area in computer vision and neuroimaging. It is usually an early step in the analysis and therefore has a major impact on the quality of the results (Despotović et al., 2015). The high performance of DL in tasks based on visual representations has led researchers to apply it to segmentation in neuroimaging. In the following paragraphs we review studies that used DL for segmentation in neuroimaging.

Ushizima et al. (2022) applied Deep Learning to the segmentation of tau proteins in PET images, which is important for understanding the neurobiological basis of AD. Their end-to-end solution was able to achieve high performance, with ROC curves ranging from 0.85 to 0.91 for different tracers. Henschel et al. (2022) developed FastSurferVINN, a voxel size independent DNN capable of performing segmentation on images with different resolutions from 0.7 to 1 mm. The proposed method outperformed state-of-the-art models for segmentation of different resolutions while overcoming the data imbalance problem. Zhao (2019) constructed a Bayesian DNN for brain extraction that can generate uncertainty maps for each pixel and image. The model achieved efficiency superior to current state-of-the-art methods, was very time efficient and flexible, and could learn highly complex structures. Brown et al. (2020) demonstrated a DL approach for segmenting orbital fat, a tissue that is not usually affected by pathological processes and is therefore important for contrast normalization. The DL approach agreed with the segmentations of the adjudicating expert and performed better than the segmentations of other human experts. Billot et al. (2020) developed a fully automated segmentation method of the hypothalamus and its subunits based on T1-weighted MR scans processed by a CNN. The method, based on DL, outperformed inter-rater reliability (variability between two different raters) and approached intra-rater reliability (variability of one expert rater on two different occasions). The model also outperformed an automated multi-atlas approach and was able to generalize its segmentation ability to a larger and more heterogeneous data set (ADNI) and show sensitivity to AD-specific atrophy. Balboni et al. (2022) presented a DL model with transfer learning for hippocampal segmentation in patients with MCI and AD. The results showed very high similarity to an expert. The high precision of the model could facilitate the detection of minor abnormalities already present in MCI and thus contribute to early diagnosis. Addressing the problem of generalizing segmentation methods, Mojiri Forooshani et al. (2022) presented a Bayesian 3D convolutional neural network that can automatically segment white matter hyperintensity and output uncertainty estimates for quality control. The model was robust to different acquisition protocols and therefore had higher ability to generalize. Li et al. (2021) presented a DL-based segmentation method for the claustrum—a subcortical unit that is usually difficult to segment using classical methods—and showed equivalent or performance superior to inter-rater reliability of human experts.

## 4. Challenges and solutions

The goal of the following section is to identify common challenges and limitations faced by users of DL and propose workable solutions. We discuss challenges related to data, overfitting and regularization, architectures and hyperparameters, and computational costs.

### 4.1. Data

#### 4.1.1. Multidimensionality—space and time

Neuroimaging data can provide information in two dimensions: spatial and temporal. In some applications, such as the study of brain anatomy and structural changes, we focus primarily on the high-precision spatial information provided by sMRI. In other applications, e.g., studying the precise timing of neuronal events, oscillations, or synchrony in neuronal activity, we may focus primarily on temporal information provided by high temporal and low spatial resolution methods such as EEG and MEG. In many cases, though, such as the study of brain function, it is important to consider both spatial and temporal information. Furthermore, when using neuroimaging data to discriminate between individuals or groups, it is beneficial to consider both brain structure and brain function together. For example, it is well-known that both functional connectivity and brain structure are altered in psychiatric disorders. The optimal solution in these cases is to combine or merge the information from the two dimensions. To achieve this, an architecture that can incorporate both dimensions should be applied with a model or combination of models that can handle spatial and sequential data.

The first approach that comes to mind is the most popular: a combination of CNNs for spatial processing and RNNs for temporal processing. This method has been tested several times in neuroscience research in recent years. Dakka et al. (2017) applied a combination of CNN and RNN to 4D fMRI data to distinguish patients with schizophrenia from healthy controls. A CNN extracted spatial information, which was then sent to an RNN whose output was a binary classification. The architecture achieved 64.9% accuracy, which was better than the performance of SVM but worse than that of a global functional connectivity model.

Yan et al. (2019) combined a CNN and an RNN to create a multiscale RNN that could process spatiotemporal data. The input was time components (TCs) from different spatial components (ICs) extracted with Independent Component Analysis (ICA). The convolutional layers had filters of different sizes that allowed the model to analyze the data at multiple time scales. The authors reported 83.4% accuracy, outperforming other comparable models (AdaBoost, random forest, SVM).

Hebling Vieira et al. (2021) used an ensemble of RNNs to predict general intelligence (g-factor), feeding time series of 360 ROIs into the model. Using their approach, they found networks that predicted g-factor better than other resting state networks of similar size. Similarly, Wang (2020) developed a new DL architecture for analyzing fMRI data. The convolutional RNN, consisted of convolutions to extract spatial features in ROIs and an RNN to process the temporal aspect of the data. The convolutional RNN outperformed the conventional RNN on most single-subject identification tasks with different window sizes (number of frames). The convolutional layers also facilitated visualization of important features.

Mao et al. (2019) developed a DL architecture that used 3D CNNs to extract spatial features from each fMRI frame and passed these latent features to an RNN to process temporal dependencies within task-evoked brain activity, and achieved 71.3% accuracy in ADHD diagnosis. Wang et al. (2020b) developed a CNN in which the first convolutional layer was able to generate temporal descriptors for each voxel. The model, trained for seven-fold classification of brain states, was able to achieve an impressive accuracy of 93.7%, which is about 25% higher than the accuracy obtained with the combination of MVPA and SVM. In addition, the model was successfully fine-tuned on smaller data sets for predicting subtypes of working memory and motor tasks. Jiang et al. (2022), working with the same data set, used four-dimensional kernels to process time series of fMRI data, which were then flattened and sent to the attention module working in parallel with feature extraction. The accuracy of the model in decoding brain state was 97.4%. Supekar et al. (2022) trained a spatiotemporal CNN to discriminate between male and female ASD patients and achieved 86% prediction accuracy. Kashef (2022) constructed a CNN with blocks of temporal convolutional layers using normal convolutions and dilations, giving the model a large temporal receptive field. The model achieved 80% accuracy in diagnosing ASD. D'Souza et al. (2021) presented a deep generative hybrid approach. Instead of static, they used dynamic functional connectivity matrices that measure synchrony between regional time series as a function of time. Factorizations of these matrices were regularized by a structurally regularized dynamic dictionary learning module and decomposed into time-varying subject-specific loadings that were used as inputs to an RNN to predict clinical scores. In this way, they were able to capture subject-specific and group-based information and outperform several state-of-the-art methods.

Another modality that poses a similar problem (and solution) is EEG. Like fMRI, EEG can carry spatial and temporal information, although the former is much coarser and the latter much more precise than in fMRI. According to Craik et al. (2019), solutions for data formulations in DL methods in EEG research can be divided into three categories: raw data (e.g., Fan et al., 2021; Thanjavur et al., 2021), computed features (e.g., Wang et al., 2020a; Bagherzadeh et al., 2022), and images (e.g., Loh et al., 2022). The structure of the EEG data allows it to be provided to the model as a 2D matrix, with one dimension representing electrodes and the other representing time points. Spatial and temporal convolutions can then be applied together or separately (Borra et al., 2021). Temporal dependencies can also be analyzed by RNNs, which is why some authors have combined them with CNNs (e.g., Thodoroff et al., 2016).

There are also alternative methods to extract the temporal (functional) information without requiring a sequence processing architecture. However, these methods require some preprocessing of the data, which may result in information loss. In this case, the preprocessing aims to transform the raw time series into static data compatible with models that are not capable of processing sequential data (e.g., CNNs). In other words, these methods aim to select the relevant static features of the data. This goal can be achieved using a number of methods, the most common of which are data-driven (e.g., ICA, Canonical Correlation Analysis—CCA) and seed-based (e.g., seed-based correlation) (Yan et al., 2019). The output of feature selection methods is a “time-sequence-reduced” value set, where the temporal dimension is used in a way that is based on a specific hypothesis, e.g., that the temporal dimension contains information about functional connectivity. Most feature selection methods result in functional connectivity matrices and subject-specific spatial maps (Yan et al., 2019). Since this type of data consists of multiple arrays but is not sequential, it can be easily used with CNNs. Many studies have applied DL to resting state functional connectivity fMRI data (e.g., Kim et al., 2016; Guo et al., 2017; Heinsfeld et al., 2018; Shao et al., 2021), usually achieving high levels of success. Deshpande et al. (2015) compared linear measures of functional connectivity with non-linear and causal directed measures and found that the latter did not perform better. Some authors have also experimented in the frequency domain, such as with ALFF maps, ReHo maps (Zou et al., 2017), and max-pooling after Fast Fourier Transform, which is reported to be more informative (Kuang and He, 2014). Other examples of the use of temporal data include the task-based fMRI studies mentioned earlier. In this case, the “task condition” reduces the temporal dimension by itself.

#### 4.1.2. Multimodality

There are many different modalities of neuroimaging that differ in terms of the information they carry. So far, we have looked at studies using a single modality that carries spatial and temporal information (e.g., fMRI). But what if we want to combine data from multiple modalities that provide complementary information (e.g., MRI and PET, or sMRI and EEG)? This is challenging because neuroimaging modalities differ in many features and are sometimes incompatible. We have identified several possible approaches to the modality problem, which we can broadly divide into two categories: feature-based and model-based. The goal of both approaches is the same: to concatenate or fuse different types of data. If the data are compatible, vectors from different modalities can be easily merged at the model input stage (e.g., Liu et al., 2017). However, this largely ignores the complex and highly abstract relationships between different modalities (Shi et al., 2018). An alternative is to extract features from each modality separately and then combine them into a single model, a technique referred to as “feature-based fusion” (Ulloa et al., 2018). In this approach, features are extracted from each modality so that the feature representations of the data are compatible with each other and can be inserted into a single model.

In comparison, the model-based approach aims to incorporate different modalities by constructing pipelines in which multiple models operate in parallel and process different types of data. The outputs of these models are then fed to a common module that processes all the information and makes predictions. Note that the results of these DL models are extracted features.

The main difference between the feature-based approach and the model-based approach is the method and stage at which the features are extracted. While in the feature-based approach the features are extracted in the preprocessing step and the fusion step starts early, in the model-based approach the feature extraction is integrated into the DL model and the data is merged in the deeper parts of the model.

The feature-based approach includes combining ALFF maps (fMRI) with sMRI data (Ulloa et al., 2018), patch volumes (sMRI) with mean metabolic activities (FDG-PET) (Lu et al., 2018), gray matter (GM), white matter (WM), and cerebrospinal fluid (CSF) (sMRI) with ReHo maps, ALFF maps and VMH connectivity (Zou et al., 2017), GM and deformation magnitude with segmentation features (Chen et al., 2015), GM (sMRI) with CMRIGlc (PET) (Liu et al., 2014, 2015), GM and CSF (sMRI) with PET intensities (Suk et al., 2014), GMV (sMRI) with diffusion tensor imaging (DTI) features and resting-state functional connectivity features (FA, MD, ReHo, ALFF) (Niu et al., 2020), GMV (sMRI) with single nucleotide polymorphism (genetic) (Chen et al., 2021).

In a model-based approach, Shi et al. (2018) used two Deep Polynomial Networks (DPNs) to extract features from sMRI and PET, which were then combined in a third DPN to make predictions for SCZ. Gao and Hui (2016) used a 2D and 3D CNN to extract features from 2D and 3D data from CT, respectively. These features were then fused to predict AD and lesions. Zhou et al. (2021) used a CNN to extract features from PET images and then linked them to clinical parameters to predict AD. Ren et al. (2022) constructed a multimodal compact bilinear fusion module to fuse features from sMRI images and blood parameters in a CNN to predict brain age. Spasov et al. (2019) constructed a multimodal feature extractor, a model for parallel processing and concatenation of MRI and Jacobian Determinant images and clinical features. Zhou et al. (2019) proposed a three-stage process for combining sMRI, PET, and genetic modalities. In the first stage, DL was used to extract features from each modality separately, while in the second stage, the results from the first stage were used to combine pairs from the three different modalities. In the third stage, predictions were made about MCI and AD. Akada et al. (2021) found that a multimodal approach in which EEG and electromyography (EMG) data were first processed separately and then combined gave better results than a rule-based integration approach and an ensemble stacking approach.

#### 4.1.3. Imaging at multiple sites

The small size of many neuroimaging data sets is detrimental to the statistical power and proper functioning of the DL methods. The number of subjects may increase greatly when data collected at different sites are pooled. Data sharing is promising, but it also comes with limitations. Pooling data from different imaging sites not only increases biologically relevant variance, but also magnifies the effect of biologically irrelevant variance—noise—due to different conditions and technical characteristics of the equipment at each site. Dinsdale et al. (2021) proposed a DL-based solution to this challenge. Their method consists of three steps: (i) extracting features from the input data and optimizing the classifier for the target task (classification/regression/segmentation), (ii) optimizing the domain classifier for scanner classification, and (iii) using this information to optimize the feature extractor to confuse the domain classifier to remove irrelevant variance. The proposed model was able to learn a scanner-invariant feature representation while successfully performing the target task. The authors also showed that the model can be easily adapted to remove continuous and categorical confounds and can be applied to any DL architecture.

### 4.2. Overfitting and regularization

Overfitting, a key challenge in DL, is largely the result of the dimensionality curse, a property of training data that typically consists of a large number of dimensions and a small number of samples. A DL with millions of parameters can learn to perform almost perfectly on the (small) data set in question, but is unable to generalize to samples outside the training data set. Because neuroimaging data sets are typically small, while inherently complex and high-dimensional, overfitting is a major challenge to the utility of DL. Fortunately, many techniques have been developed in computer science to solve the problem of overfitting. These strategies, which aim to reduce the generalization error but not the training error, are collectively referred to as regularization methods and usually involve a constraint or penalty on internal parameters of the model (Goodfellow et al., 2016). In the next sections we present a number of commonly used regularization approaches.

#### 4.2.1. Parameter norm penalties

Regularization strategies that aim to limit the capacity of the model by adding a penalty term to the objective function are called parameter norm penalties. One of the simplest and most commonly used is the *L*_2_ or *weight decay* penalty, which constrains the weights closer to the origin by adding a penalty term to the objective function (see Equations 4 and 5). The *L*_1_ norm also adds a penalty term to the objective function, but it is computed differently. While the *L*_2_ norm is calculated as the square root of the sum of the squared vector values, the *L*_1_ norm is calculated as the sum of the absolute values of the vector. The *L*_1_ is a sparse norm, i.e., it assigns zero values to some parameters and therefore it also functions as a feature selection mechanism (Goodfellow et al., 2016).


(4) $$\begin{array}{l} J\left(\theta \right)=\;\frac{1}{N}\overset{N}{\underset{i=1}{\sum }}{\left(h_{\theta }{\left(x_{i}\right)-y}_{i}\right)}^{2}+L_{2} \end{array}$$

**Equation 4**. Loss function with the *L*_2_ penalty norm.


(5) $$\begin{array}{l} L_{2}=\lambda \overset{M}{\underset{j=1}{\sum }}\theta_{j}^{2} \end{array}$$

**Equation 5**. *L*_2_ penalty norm. The effect of the penalty norm can be regulated *via* adjusting the λ.

#### 4.2.2. Data augmentation

The optimal solution to overfitting would simply be to have more data available. If the data are in a format where many of their properties can be easily manipulated, this can be achieved by simply transforming the samples. Data augmentation is particularly useful for images, for which numerous transformation tools are available. Data augmentation can improve generalizability (Goodfellow et al., 2016), but has limited potential because the distribution of augmented samples is usually similar to that of the original samples (Lan et al., 2020). An alternative could be not to apply simple transformations to images, such as rotating, scaling, and cropping, but to apply complex transformations by using DL generative models such as GANs, which are known to perform very well in data synthesis. Applications of GANs in neuroimaging are discussed in *Generation*.

Wang et al. (2018) proposed a DL model to classify patients with alcoholism based on sMRI. Since only 235 sMRI images were available, they attempted to improve the generalization ability by augmentation. Image augmentation resulted in 13100 images for their final training set, which allowed them to achieve 97% accuracy. Data augmentation has also been used by Wang et al. (2017) to identify MCI based on sMRI images and by Zou et al. (2017) to diagnose ADHD based on sMRI and fMRI data. Olawunmi Olaboopo (2021) applied augmentation to EEG data to decode motor imagery. An interesting application of augmentation was also presented by Wang et al. (2020b), who augmented fMRI time series in the temporal dimension.

#### 4.2.3. Semi-supervised learning

Semi-supervised learning refers to the use of both an unsupervised model for learning how the data is distributed or clustered in a low-dimensional space and a supervised model for classification. The two parts can be done either independently or together, so that the two models share their parameters. In this way, it is easier to find the optimal tradeoff between the two models (Goodfellow et al., 2016).

Semi-supervised learning has been widely used in neuroimaging research. Examples include the use of AEs (Guo et al., 2017) and DBNs (Jang et al., 2017) with deep discriminant models. Guo et al. (2017) found that AEs can achieve better accuracy than other unsupervised models (*t*-test and elasticnet). However, most authors did not compare semi-supervised learning with supervised learning, which makes it difficult to draw definitive conclusions.

#### 4.2.4. Multitask learning

In multitask learning, a model consists of a generic part with shared parameters and task-specific parts with independent weights that perform the target task. The basic idea is that sharing input and intermediate representations constrains the parameters toward better generalization (Goodfellow et al., 2016). For example, Liu et al. (2017) showed that a DL model with two tasks, a classification task and a clinical score regression task, performed better than a model with a single task.

#### 4.2.5. Early stopping

In DL training, it is common that beyond a certain point, validation accuracy reaches a plateau or even decreases while training accuracy continues to improve. The increasing difference between training and validation accuracy indicates that the model is starting to overfit. Therefore, the training should be terminated early. Early stopping is a rule that instructs the model to stop training if the validation accuracy has not improved for a certain number of iterations (Goodfellow et al., 2016).

#### 4.2.6. Parameter sharing

In some tasks, we know that the parameters depend on each other. For example, in images, pixels that are close to each other are usually similar. It is possible to enforce equality of a set of parameters, which has a double advantage: better generalization and lower computational cost. Parameter sharing is commonly used in CNNs. CNNs are usually used for visual tasks, where their training data are natural images that have many statistical properties that are invariant to translation. This means that an object is semantically the same regardless of where on the image it appears. CNNs are robust to object's position because they share parameters across different locations in the image (Goodfellow et al., 2016). Like other natural images, brain images have translation-invariant statistical properties (e.g., the hippocampus can be observed at different locations in images taken from different angles), making parameter sharing an appropriate strategy.

#### 4.2.7. Sparse representations

Sparse representation penalty is somewhat similar to the *L*_1_ norm penalty in that both enforce zero values in the model. However, while the *L*_1_ norm introduces sparse parametrization by nullifying sets of parameters, sparse representation sets the elements in the representation vector (Goodfellow et al., 2016) to zero.

Suk et al. (2017) combined sparse regression models that learned feature representations with CNN that performed diagnosis of MCI and AD. The DL solution was able to outperform linear classifiers. Chen et al. (2021) used a sparse DNN for better feature interpretability in diagnosing SCZ. The results showed that the sparse DL method was able to fuse neuroimaging and genetic features better than the combination of ICA and SVM.

#### 4.2.8. Ensemble methods

In the ensemble method, several models are trained independently and then an average is taken over all models. This method helps with generalization because the models trained separately do not make the same errors. If the errors they make are uncorrelated, then the expected squared error of the ensemble is inversely proportional to the ensemble size. In theory, the performance of an ensemble is at least as good as that of a single model. Ensemble training is a powerful method for overcoming overfitting, but it comes at a price: it is very computationally intensive (Goodfellow et al., 2016).

Several authors have proposed ensemble solutions for neuroimaging analysis. Lu et al. (2018) presented ensemble training using a multiscale multimodal approach. They trained six different DNNs with three different scales and two modalities (MRI and PET). The features of the DNNs were then fused by another DNN that performed a three-way classification (healthy controls, MCI, AD) with higher success than other comparable models. Hebling Vieira et al. (2021) used an ensemble of RNNs to limit variance in predicting general intelligence based on time-series fMRI data.

#### 4.2.9. Dropout

**Figure 8:**
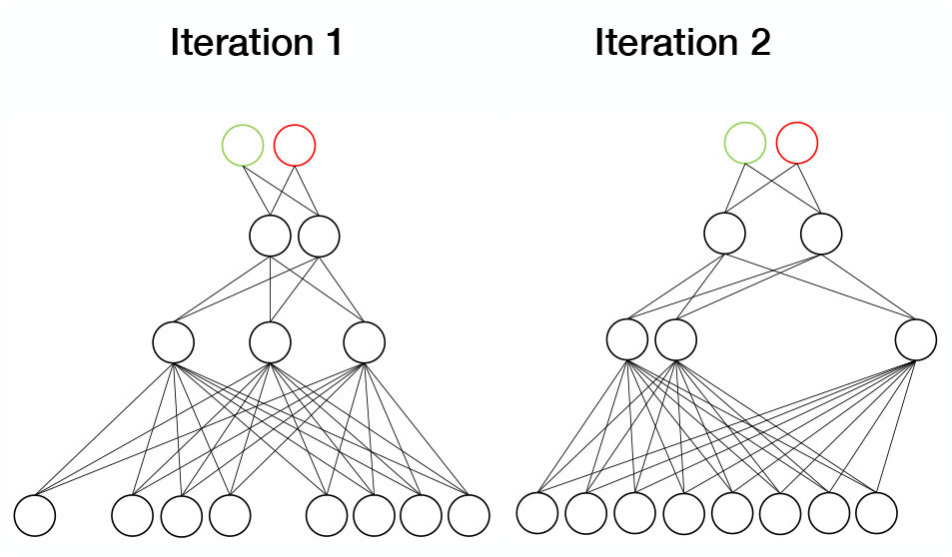
Dropout. In each sample, a different set of weights are set to zero. Since each iteration then involves a different subnetwork, dropout simulates ensemble training.

Dropout (Figure 8) refers to annulling a proportion of the model parameters by multiplying them with zeros. In each sample, a different set of parameters is chosen. Dropout thus represents a computationally inexpensive approximation to ensemble training, because by annulling different parameter combinations, an ensemble of subnetworks is essentially created. Typically, the dropout rate is 0.2 for the visible parameters and 0.5 for the hidden parameters (Goodfellow et al., 2016). Shen et al. (2020) used the dropout strategy in their deep polynomial model to increase generalization in PD diagnosis. They were able to achieve a prediction accuracy of 86%.

#### 4.2.10. Transfer learning

**Figure 9:**
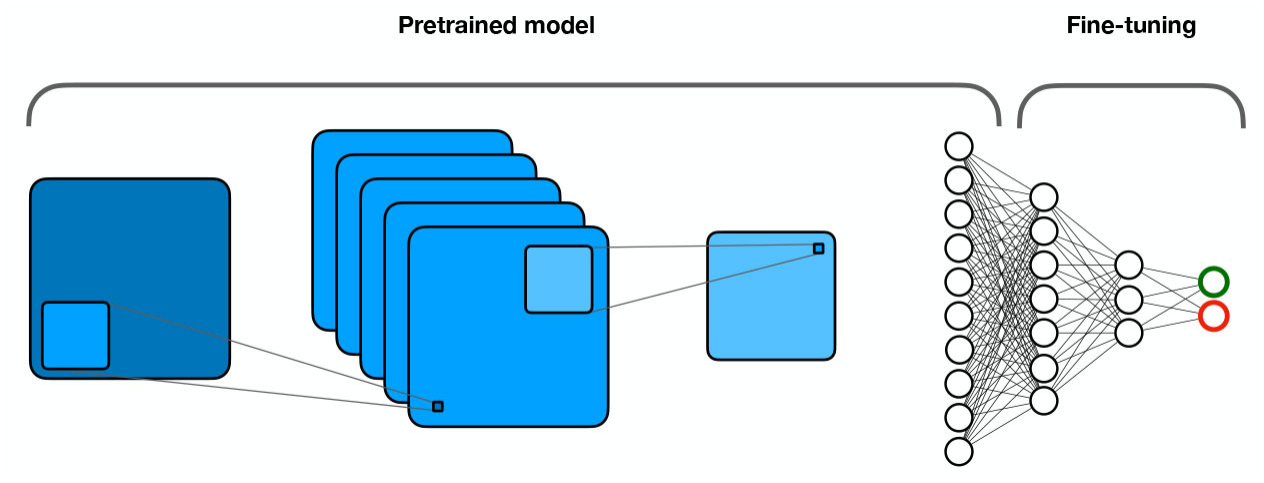
Transfer learning. Part of the model is pretrained on a larger data set, while the other part is fine-tuned on the target dataset.

Transfer learning (Figure 9) is based on the hypothesis that the training data need not be independent and identical to the test data. Essentially, it refers to the transfer of knowledge from a source domain to a target domain (Tan et al., 2018). It assumes that different domains share low-level features, while high-level features are specific to each domain. Instead of training a model from scratch, transfer learning proposes to reuse the weights of a trained model in the lower layers and fine-tune only the weights in the higher layers with the data from the target domain. This is particularly useful for tasks where only small amounts of data are available for the target domain. It also significantly reduces computational costs.

Numerous authors have suggested using transfer learning to increase generalization. Heinsfeld et al. (2018) used features learned from stacked AEs to fine-tune a perceptron for ASD diagnosis. Hosseini-Asl et al. (2018) used 3D AEs to learn anatomical variations in the source domain from sMRI images and then fine-tuned a CNN in the target domain (AD classification), resulting in over 90% accuracy.

Based on the assumption that MRI images share statistical features with natural images, Gupta et al. (2013) extracted features from natural images with AEs and then used the learned features to refine a CNN for three-way classification (healthy controls, MCI, AD). The model achieved comparable or better accuracy than competing models. Ngo et al. (2022) trained a DL model to classify task-based resting-state fMRI activity. They found that pretraining the model with a larger data set and then fine-tuning it with a smaller data set led to significantly better prediction results than training with small data sets from scratch. They suggest that the success of transfer learning is due in part to the multitasking nature of their network. By performing multiple tasks, the model was able to learn representations that were important for different tasks. Wang et al. (2021) pre-trained a DNN on a large human data set to perform brain extraction (skull stripping) on a small sample of non-human primates. Their model performed better than other comparable models.

Dehghani et al. (2021) pretrained a model on EEG data from multiple participants and fine-tuned it to a single participant to achieve more accurate motor imagery decoding. The method was superior to models such as SVM in terms of learning and classification accuracy. Wang et al. (2020b) first trained a 3D CNN for 7-way classification based on a large task-based fMRI data set (a HCP data set with 1,034 participants). They then fine-tuned the model on two data sets with fewer than 50 participants to distinguish between two working memory and four motor tasks. The prediction accuracy was 93.7% for the general 7-way classification task and 89 and 94.7% for the WM and the motor tasks, respectively.

Jiang et al. (2022) went one step further and evaluated transferability not only to new data but also to new tasks. They pre-trained their 4D CNN model for 7-way classification using the same data set as Wang et al. (2020b). They then fine-tuned the model with a subset of the WM task and used it to regress general intelligence (gF). In the second transfer learning task, the model was fine-tuned with a visual perception data set consisting of only four participants. The task of the model was to solve a binary classification problem (object vs. scene). Their solution showed results superior to those of similar studies and to those of non-transfer conditions, with a prediction accuracy of 97.4% for the general 7-way classification task, a Spearman's correlation of 0.354 for the first transfer task, and a prediction accuracy of 77.6% for the second task. Furthermore, visualization analysis suggests that low-level attentional masks (representations) remain the same, whereas high-level attentional masks adapt to the target task in the transfer condition.

Wang et al. (2020a) tried to overcome the lack of labeled EEG data by transfer learning. They trained a CNN with electrode distribution frequency maps (EDFM) from a larger data set and then fine-tuned it on a data set with only a few samples, achieving 90.59 and 82.84% accuracy, respectively. Bagherzadeh et al. (2022) used classic CNNs (AlexNet, ResNet-50, Inception-v3, and VGG-19) pre-trained on natural images from the ImageNet data set (Deng et al., 2009) and adapted them to decode emotions from EEG data. Similarly, Helaly et al. (2021) used a pre-trained VGG-19 model and fine-tuned it to predict MCI and AD based on sMRI data. Both studies report impressive results with accuracies above 95%. Balboni et al. (2022) applied transfer learning to a hippocampal segmentation method, Spatial Warping Network Segmentation (SWANS), trained on a large AD data set (ADNI) and fine-tuned on data sets with different acquisition protocols. The transfer method outperformed the original segmentation method.

### 4.3. Architecture and hyperparameters

Choosing the architecture and hyperparameters of the model is not a simple undertaking. It depends largely on what goal we are pursuing, and usually consists of several trials with different combinations of hyperparameters until we find the optimal solution. It is worth noting that by far the most popular architecture of DL in neuroimaging studies have been CNNs and their subtypes. This is not very surprising considering that they excel in visual representation tasks, of which neuroimaging is essentially one. Vieira et al. (2017) reported that CNNs (Figure 4A) and combinations of CNNs with AEs performed better than sole AE applications. Indeed, CNNs are applicable to a wide range of tasks that are important in neuroimaging analysis. CNNs can segment and predict. They also learn representations that are interpretable and can contribute to the understanding of brain mechanisms. In their classical form, CNNs are not able to process time series data. However, several solutions to this problem have been proposed using either spatiotemporal convolutions (Wang et al., 2020b; Jiang et al., 2022; Kashef, 2022; Supekar et al., 2022) or RNNs (Figure 4B) (Dakka et al., 2017; Yan et al., 2019; Wang, 2020). One thing that CNNs cannot do is generation. The problem of data generation is the domain of AEs (Figure 4C) and GANs (Figure 5). Some interesting adaptations of DNNs have been proposed. Deep polynomial networks that attempt to learn polynomial predictors within a DL architecture are said to be particularly suitable for small sample sizes (Shi et al., 2018). Another promising approach is to extend the model to include an attentional mechanism that enhances the discriminative representations of an object, making them more interpretable (Jiang et al., 2022).

An even more difficult task is the choice of hyperparameters (hyperparameter optimization—HPO). HPO is largely based on experience with DL training, which usually leads to a useful but not optimal choice (Yu and Zhu, 2020). Given the large impact of hyperparameters on training results, automated HPO strategies have recently received some attention. These strategies aim to reduce the manual workload and increase the effectiveness and reproducibility of training programmes. Essentially, HPO refers to the process of finding a combination of hyperparameters that results in the lowest possible loss and the highest possible accuracy of the network. It can be divided into two categories: search algorithms and trial schedulers. The former aim to sample different combinations of hyperparameters, while the latter deal with early stopping and model evaluation (Yu and Zhu, 2020).

### 4.4. Computational cost

VGG-16, one of the most successful and widely used CNN architectures, has about 138 million learnable parameters (Simonyan and Zisserman, 2014). In practice, this means that training the mammoth structure requires extensive computational resources and time. Indeed, convergence of such a model on a powerful GPU typically takes several hours to several days. One of the main reasons for the popularity of DL in recent years has been the development of powerful computer hardware. Without fast computers, it could take months for such models to converge. In absence of significant increase in computational efficiency, further progress of DL is economically, technically, and environmentally unsustainable. There is an urgent need to improve computational performance by either making changes to DL or switching to other machine learning methods (Thompson et al., 2020). Here we would like to discuss possible modifications to DL. One possibility is to use lightweight architectures (Borra et al., 2021), i.e., models with a smaller number of parameters. This solution seems to be suitable for neuroimaging applications, since we usually deal with small data samples where large models can easily be overfitted. Reducing the number of parameters can also be achieved by regularization strategies such as dropout (Figure 8) and sparsity norms. In this way, we can kill two birds with one stone: the problem of computational cost and overfitting.

## 5. Discussion

In this review, we examined the most common applications of DL in neuroimaging data analysis, their challenges, and possible solutions. Prediction, one of the hallmarks of DL, has been applied very successfully to neuroimaging data and holds great potential for the future. It is worth noting that combining multiple modalities usually yields better results than using only a single modality. Certainly, the relationships between different types of data can be highly complex and abstract, which fits perfectly with the logic of DL. Moreover, 3D models have been shown to perform better than models with lower dimensions. On the one hand, the explanation for the higher performance is simple: more dimensions mean more relevant information. On the other hand, this is also indicative of the nature of the DL models, which seem to be quite capable of processing data in its rawest form. Based on this observation it would be counterintuitive to preprocess the data in a way that leads to a loss of information.

Although the DL models perform well on data from which features have been extracted prior to training, the preprocessing step appears to be unnecessary or even potentially harmful. Indeed, many of the feature extraction techniques impose *a priori* hypotheses, which move DL away from a fully data-driven approach. From this point of view, prior feature extraction can be seen as a limitation that prevents DL from exploiting the richness of information in the raw data. On the other hand, the rawness raises other problems, most notably the high dimensionality. This is not a problem *per se*, but becomes a risk when data is scarce. As mentioned, high dimensional data combined with few samples are prone to overfitting. In addition, unprocessed data is much more computationally intensive. Nonetheless, there have been very successful applications of DL to raw data. The encouraging results from researchers who have developed models capable of processing spatial and temporal data simultaneously are promising for the future. In the study by Jiang et al. (2022), whose model was able to achieve 97.4% accuracy on a 7-way classification task, the success of the model was attributed to 4D (spatiotemporal) kernels that allowed it to jointly process dynamic changes and integrate them with interconnected brain regions. In addition, performance was improved and training time was significantly reduced (from 19 to 12 h) by a 3D attention mechanism that was able to adaptively allocate processing focus.

Overfitting and computational costs can also be addressed together with some regularization strategies. Sparsity norms annihilate parameters or parts of representations, which simultaneously reduces dimensionality and computational cost. Dropout (Figure 8) nullifies a random combination of nodes in each iteration, making the computational process more time efficient while mimicking ensemble training, which can improve generalization. Transfer learning (Figure 9) also holds great potential and has been shown to improve generalizability. Moreover, it works not only with source data sets that are semantically related to neuroimaging, but also with other natural images, such as those in the Imagenet data set (Deng et al., 2009). Given that DL platforms such as Keras offer many pre-trained models, this could significantly reduce training time.

The question remains how far knowledge can be transferred. Given the strong comorbidity of psychiatric disorders, it has been argued that a general psychopathology factor (p-factor) exists that can explain the variance between different disorders (Gluschkoff et al., 2019). Based on this assumption, new avenues of research can be proposed for DL. One possible example would be a learning system in which pretraining would include data from patients with different disorders, possibly from different data modalities, while fine-tuning would consist of the target disorder. While DL has been widely applied in the study of some disorders such as SCZ, ASD, and AD, some others such as obsessive-compulsive disorder, depression, and anxiety disorders have been ignored despite their high prevalence. Moreover, to support the idea of initiatives such as RDoC (Insel et al., 2010), the task need not be to diagnose a psychiatric disorder but to predict a refined (less heterogeneous) criterion variable, such as symptom, severity, or location on a spectrum. Indeed, such proposals have already been made (Sheynin et al., 2021).

In addition to overcoming the problem of overfitting, generative models offer other interesting possibilities for scientific work. The presumed ability of GANs to represent the entire distribution of data could be fruitful in the study of disorder spectra. Training GAN to generate neuroimaging data from data of patients with different disorders that form a spectrum (e.g., SCZ, schizoaffective disorder, and BD) might allow us to examine in detail the subtle changes in neuronal structure and/or function by moving through the latent representational space. External validity of the generated data is also of great importance. This could be tested by training a model with synthetic data and testing it on real data.

The success of neuroscientific research using DL depends largely on the ability to interpret the internal mechanism of a model. Therefore, the quality of the results depends on the level of sophistication of the interpretative methods. As mentioned earlier, the fusion of Guided Back-propagation and Grad-CAM provides a class-discriminative high-resolution method—a perfect match for DL applications in neuroimaging. Undoubtedly, as Deep Learning advances, new, increasingly sophisticated methods of XAI will be developed. Of course, we should only consider interpretations of models that we trust. That is, models that score well on an evaluation metric (e.g., have high test accuracy).

Our decisions about architecture and hyperparameters have a large impact on the final product. Therefore, our decisions should be supported by a logical and empirical rationale. The choice of architecture depends on the task that the model is intended to perform. As for the hyperparameters, automated selection procedures have been developed but are rarely used in neuroimaging applications (e.g., Treacher et al., 2021).

Finally, a unified working framework for DL in neuroimaging could facilitate communication and exchange of ideas and practical solutions among neuroscientists. Kuntzelman et al. (2021) have developed a Python software toolbox, DeLINEATE, that is specifically designed to facilitate neuroscience research using deep multivariate pattern analysis (dMVPA). Its main function is to enable scientists to explore different architectures and hyperparameters and compare their performance with each other and with other (non-deep) methods. DeLINEATE is an ongoing project and we can expect future developments (new architectures, transfer learning, visualization techniques) that will provide researchers with even more flexibility and sophistication in DL neuroimaging applications.

## Author contributions

LA and GR contributed to conception and outline of the article. LA reviewed the literature and wrote the first draft of the manuscript. Both authors reviewed, edited, and contributed to the final version of the manuscript. Both authors read and approved the submitted version.

## Funding

This work was supported by the Slovenian Research Agency research Grants P3-0338 and J3-9264 (GR).

## Conflict of interest

Author GR consults for and holds equity in Neumora Therapeutics and Manifest Technologies. The remaining author declares that the research was conducted in the absence of any commercial or financial relationships that could be construed as a potential conflict of interest.

## Publisher's note

All claims expressed in this article are solely those of the authors and do not necessarily represent those of their affiliated organizations, or those of the publisher, the editors and the reviewers. Any product that may be evaluated in this article, or claim that may be made by its manufacturer, is not guaranteed or endorsed by the publisher.

## References

[B1] Abdul Nabi Ali A. Alam M. Klein S. C. Behmann N. Krauss J. K. Doll T.. (2022). Predictive accuracy of CNN for cortical oscillatory activity in an acute rat model of Parkinsonism. Neural Netw. 146, 334–340. 10.1016/j.neunet.2021.11.025 34923220

[B2] Abou Jaoude M. Sun H. Pellerin K. R. Pavlova M. Sarkis R. A. Cash S. S.. (2020). Expert-level automated sleep staging of long-term scalp electroencephalography recordings using deep learning. Sleep 43, zsaa112. 10.1093/sleep/zsaa112 32478820 PMC7686563

[B3] Akada K. Yagi T. Miura Y. Beuckmann C. T. Koyama N. Aoshima K. (2021). A deep learning algorithm for sleep stage scoring in mice based on a multimodal network with fine-tuning technique. Neurosci. Res. 173, 99–105. 10.1016/j.neures.2021.07.003 34280429

[B4] Bach S. Binder A. Montavon G. Klauschen F. Müller K.-R. Samek W. (2015). On pixel-wise explanations for non-linear classifier decisions by layer-wise relevance propagation. PLoS ONE 10, e0130140. 10.1371/journal.pone.0130140 26161953 PMC4498753

[B5] Bae J. Stocks J. Heywood A. Jung Y. Jenkins L. Hill V.. (2021). Transfer learning for predicting conversion from mild cognitive impairment to dementia of Alzheimer's type based on a three-dimensional convolutional neural network. Neurobiol. Aging 99, 53–64. 10.1016/j.neurobiolaging.2020.12.005 33422894 PMC7902477

[B6] Bagherzadeh S. Maghooli K. Shalbaf A. Maghsoudi A. (2022). Emotion recognition using effective connectivity and pre-trained convolutional neural networks in EEG signals. Cogn. Neurodyn. 16, 1087–1106. 10.1007/s11571-021-09756-0 36237402 PMC9508317

[B7] Balboni E. Nocetti L. Carbone C. Dinsdale N. Genovese M. Guidi G.. (2022). The impact of transfer learning on 3D deep learning convolutional neural network segmentation of the hippocampus in mild cognitive impairment and Alzheimer disease subjects. Hum. Brain Mapp. 43, 3427–3438. 10.1002/hbm.25858 35373881 PMC9248306

[B8] Barile B. Marzullo A. Stamile C. Durand-Dubief F. Sappey-Marinier D. (2021). Data augmentation using generative adversarial neural networks on brain structural connectivity in multiple sclerosis. Comput. Methods Prog. Biomed. 206, 106113. 10.1016/j.cmpb.2021.106113 34004501

[B9] Bhardwaj H. Tomar P. Sakalle A. Ibrahim W. (2021). EEG-based personality prediction using fast fourier transform and DeepLSTM model. Comput. Intell. Neurosci. 2021, 1–10. 10.1155/2021/6524858 34603433 PMC8481053

[B10] Billot B. Bocchetta M. Todd E. Dalca A. V. Rohrer J. D. Iglesias J. E. (2020). Automated segmentation of the hypothalamus and associated subunits in brain MRI. Neuroimage 223, 117287. 10.1016/j.neuroimage.2020.117287 32853816 PMC8417769

[B11] Borra D. Fantozzi S. Magosso E. (2021). A lightweight multi-scale convolutional neural network for P300 decoding: analysis of training strategies and uncovering of network decision. Front. Hum. Neurosci. 15, 655840. 10.3389/fnhum.2021.655840 34305550 PMC8295472

[B12] Brosch T. Tam R. (2013). “Manifold learning of brain MRIs by deep learning,” in International Conference on Medical Image Computing and Computer-Assisted Intervention (Berlin, Heidelberg: Springer), 633–640. 10.1007/978-3-642-40763-5_78 24579194

[B13] Brown R. A. Fetco D. Fratila R. Fadda G. Jiang S. Alkhawajah N. M.. (2020). Deep learning segmentation of orbital fat to calibrate conventional MRI for longitudinal studies. Neuroimage 208, 116442. 10.1016/j.neuroimage.2019.116442 31821865

[B14] Chen J. Li X. Calhoun V. D. Turner J. A. Erp T. G. M. Wang L.. (2021). Sparse deep neural networks on imaging genetics for schizophrenia case-control classification. Hum. Brain Mapp. 42, 2556–2568. 10.1002/hbm.25387 33724588 PMC8090768

[B15] Chen Y. Shi B. Smith C. D. Liu J. (2015). Nonlinear feature transformation and deep fusion for Alzheimer's disease staging analysis,” in Machine Learning in Medical Imaging, eds L. Zhou, L. Wang, Q. Wang, and Y. Shi (Cham. Springer International Publishing), 304–312. 10.1007/978-3-319-24888-2_37

[B16] Craik A. He Y. Contreras-Vidal J. L. (2019). Deep learning for electroencephalogram (EEG) classification tasks: a review. J. Neural Eng. 16, 031001. 10.1088/1741-2552/ab0ab5 30808014

[B17] Dakka J. Bashivan P. Gheiratmand M. Rish I. Jha S. Greiner R. (2017). Learning neural markers of schizophrenia disorder using recurrent neural networks. arXiv preprint arXiv:1712.00512. 10.48550/arXiv.1712.00512

[B18] Dehghani M. Mobaien A. Boostani R. (2021). A deep neural network-based transfer learning to enhance the performance and learning speed of BCI systems. Brain Comput. Interfaces 8, 14–25. 10.1080/2326263X.2021.1943955

[B19] Deng J. Dong W. Socher R. Li L.-J. Li K. Fei-Fei L. (2009). “ImageNet: a large-scale hierarchical image database,” in 2009 IEEE Conference on Computer Vision and Pattern Recognition (Miami, FL), 248–255. 10.1109/CVPR.2009.5206848

[B20] Deshpande G. Wang P. Rangaprakash D. Wilamowski B. (2015). Fully connected cascade artificial neural network architecture for attention deficit hyperactivity disorder classification from functional magnetic resonance imaging data. IEEE Trans. Cybern. 45, 2668–2679. 10.1109/TCYB.2014.2379621 25576588

[B21] Despotović I. Goossens B. Philips W. (2015). MRI segmentation of the human brain: challenges, methods, and applications. Comput. Math. Methods Med. 2015, 1–23. 10.1155/2015/450341 25945121 PMC4402572

[B22] Dinsdale N. K. Jenkinson M. Namburete A. I. (2021). Deep learning-based unlearning of dataset bias for MRI harmonisation and confound removal. Neuroimage 228, 117689. 10.1016/j.neuroimage.2020.117689 33385551 PMC7903160

[B23] Dong M. Xie L. Das S. R. Wang J. Wisse L. E. deFlores R.. (2021). DeepAtrophy: teaching a neural network to detect progressive changes in longitudinal MRI of the hippocampal region in Alzheimer's disease. Neuroimage 243, 118514. 10.1016/j.neuroimage.2021.118514 34450261 PMC8604562

[B24] D'Souza N. Nebel M. Crocetti D. Robinson J. Wymbs N. Mostofsky S.. (2021). Deep sr-DDL: deep structurally regularized dynamic dictionary learning to integrate multimodal and dynamic functional connectomics data for multidimensional clinical characterizations. Neuroimage 241, 118388. 10.1016/j.neuroimage.2021.118388 34271159 PMC8528511

[B25] Durstewitz D. Koppe G. Meyer-Lindenberg A. (2019). Deep neural networks in psychiatry. Mol. Psychiatry 24, 1583–1598. 10.1038/s41380-019-0365-9 30770893

[B26] Ebbinghaus H. (1908). Psychology: An Elementary Text-Book. Boston, MA: D C Heath & Co Publishers. 10.1037/13638-000

[B27] Ebrahimi H. Shalbaf A. Jafarnia Dabanloo N. (2020). Classification of right and left hand motor imagery using deep learning in electroencephalography and near-infrared spectroscopy. Adv. Cogn. Sci. 22, 95–104. 10.30699/icss.22.3.95

[B28] Fan C.-C. Yang H. Hou Z.-G. Ni Z.-L. Chen S. Fang Z. (2021). Bilinear neural network with 3-D attention for brain decoding of motor imagery movements from the human EEG. Cogn. Neurodyn. 15, 181–189. 10.1007/s11571-020-09649-8 33786088 PMC7947100

[B29] Fischer A. Igel C. (2012). “An introduction to restricted Boltzmann machines,” in Progress in Pattern Recognition, Image Analysis, Computer Vision, and Applications, eds D. Hutchison, T. Kanade, J. Kittler, J. M. Kleinberg, F. Mattern, J. C. Mitchell, M. Naor, O. Nierstrasz, C. Pandu Rangan, B. Steffen, M. Sudan, D. Terzopoulos, D. Tygar, M. Y. Vardi, G. Weikum, L. Alvarez, M. Mejail, L. Gomez, and J. Jacobo (Berlin; Heidelberg: Springer), 14–36. 10.1007/978-3-642-33275-3_2

[B30] Gao X. W. Hui R. (2016). “A deep learning based approach to classification of CT brain images,” in 2016 SAI Computing Conference (SAI) (London, UK), 28–31. 10.1109/SAI.2016.7555958

[B31] Gluschkoff K. Jokela M. Rosenström T. (2019). The general psychopathology factor: structural stability and generalizability to within-individual changes. Front. Psychiatry 10, 594. 10.3389/fpsyt.2019.00594 31543833 PMC6728891

[B32] Golmohammadi M. Harati Nejad Torbati A. H. Lopez de Diego S. Obeid I. Picone J. (2019). Automatic analysis of EEGs using big data and hybrid deep learning architectures. Front. Hum. Neurosci. 13, 76. 10.3389/fnhum.2019.00076 30914936 PMC6423064

[B33] Goodfellow I. Bengio Y. Courville A. (2016). Deep Learning. Cambridge, MA: MIT Press.

[B34] Goodfellow I. J. Pouget-Abadie J. Mirza M. Xu B. Warde-Farley D. Ozair S.. (2014). Generative adversarial networks. arXiv [preprint]. arXiv:1406.2661. 10.48550/arXiv.1406.2661

[B35] Guo X. Dominick K. C. Minai A. A. Li H. Erickson C. A. Lu L. J. (2017). Diagnosing autism spectrum disorder from brain resting-state functional connectivity patterns using a deep neural network with a novel feature selection method. Front. Neurosci. 11, 460. 10.3389/fnins.2017.00460 28871217 PMC5566619

[B36] Gupta A. Ayhan M. S. Maida A. (2013). “Natural image bases to represent neuroimaging data,” in ICML (Atlanta, GA).

[B37] Han X. Zhong Y. He L. Yu P. S. Zhang L. (2015). “The unsupervised hierarchical convolutional sparse auto-encoder for neuroimaging data classification,” in Brain Informatics and Health, eds Y. Guo, K. Friston, F. Aldo, S. Hill, and H. Peng (Cham: Springer International Publishing), 156–166. 10.1007/978-3-319-23344-4_16

[B38] Hao A. J. He B. L. Yin C. H. (2015). “Discrimination of ADHD children based on Deep Bayesian Network,” in 2015 IET International Conference on Biomedical Image and Signal Processing (ICBISP 2015) (Beijing, China), 1–6. 10.1049/cp.2015.0764

[B39] Hassanpour A. Moradikia M. Adeli H. Khayami S. R. Shamsinejadbabaki P. (2019). A novel end-to-end deep learning scheme for classifying multi-class motor imagery electroencephalography signals. Expert Syst. 36, 1–21. 10.1111/exsy.12494

[B40] He C. Liu J. Zhu Y. Du W. (2021). Data augmentation for deep neural networks model in EEG classification task: a review. Front. Hum. Neurosci. 15, 765525. 10.3389/fnhum.2021.765525 34975434 PMC8718399

[B41] Hebling Vieira B. Dubois J. Calhoun V. D. Garrido Salmon C. E. (2021). A deep learning based approach identifies regions more relevant than resting-state networks to the prediction of general intelligence from resting-state fMRI. Hum. Brain. Mapp. 42, 5873–5887. 10.1002/hbm.25656 34587333 PMC8596958

[B42] Heinsfeld A. S. Franco A. R. Craddock R. C. Buchweitz A. Meneguzzi F. (2018). Identification of autism spectrum disorder using deep learning and the ABIDE dataset. Neuroimage Clin. 17, 16–23. 10.1016/j.nicl.2017.08.017 29034163 PMC5635344

[B43] Helaly H. A. Badawy M. Haikal A. Y. (2021). Deep learning approach for early detection of Alzheimer's disease. Cogn. Comput. 14, 1711–1727. 10.1007/s12559-021-09946-2 34745371 PMC8563360

[B44] Henschel L. Kügler D. Reuter M. (2022). FastSurferVINN: Building resolution-independence into deep learning segmentation methods?a solution for HighRes brain MRI. Neuroimage 251, 118933. 10.1016/j.neuroimage.2022.118933 35122967 PMC9801435

[B45] Hirte A. U. Platscher M. Joyce T. Heit J. J. Tranvinh E. Federau C. (2021). Realistic generation of diffusion-weighted magnetic resonance brain images with deep generative models. Magnet. Reson. Imaging 81, 60–66. 10.1016/j.mri.2021.06.001 34116133

[B46] Hosseini-Asl E. Ghazal M. Mahmoud A. Aslantas A. Shalaby A. M. Casanova M. F.. (2018). Alzheimer's disease diagnostics by a 3D deeply supervised adaptable convolutional network. Front. Biosci. 23, 584–596. 10.2741/4606 28930562

[B47] Hu J. Kuang Y. Liao B. Cao L. Dong S. Li P. (2019). A multichannel 2D convolutional neural network model for task-evoked fMRI data classification. Comput. Intell. Neurosci. 2019, 1–9. 10.1155/2019/5065214 32082370 PMC7012272

[B48] Hu M. Qian X. Liu S. Koh A. J. Sim K. Jiang X.. (2022). Structural and diffusion MRI based schizophrenia classification using 2D pretrained and 3D naive convolutional neural networks. Schizophr. Res. 243, 330–341. 10.1016/j.schres.2021.06.011 34210562

[B49] Insel T. Cuthbert B. Garvey M. Heinssen R. Pine D. S. Quinn K.. (2010). Research domain criteria (RDoC): toward a new classification framework for research on mental disorders. AJP 167, 748–751. 10.1176/appi.ajp.2010.09091379 20595427

[B50] Islam J. Zhang Y. (2020). GAN-based synthetic brain PET image generation. Brain Inf. 7, 3. 10.1186/s40708-020-00104-2 32232602 PMC7105582

[B51] Jang H. Plis S. M. Calhoun V. D. Lee J.-H. (2017). Task-specific feature extraction and classification of fMRI volumes using a deep neural network initialized with a deep belief network: evaluation using sensorimotor tasks. Neuroimage 145, 314–328. 10.1016/j.neuroimage.2016.04.003 27079534 PMC5064875

[B52] Jiang Z. Wang Y. Shi C. Wu Y. Hu R. Chen S.. (2022). Attention module improves both performance and interpretability of four-dimensional functional magnetic resonance imaging decoding neural network. Hum. Brain Mapp. 43, 2683–2692. 10.1002/hbm.25813 35212436 PMC9057093

[B53] Jonas S. Rossetti A. O. Oddo M. Jenni S. Favaro P. Zubler F. (2019). EEG-based outcome prediction after cardiac arrest with convolutional neural networks: performance and visualization of discriminative features. Hum. Brain Mapp. 40, 4606–4617. 10.1002/hbm.24724 31322793 PMC6865376

[B54] Jung W. Jun E. Suk H.-I. (2021). Deep recurrent model for individualized prediction of Alzheimer's disease progression. Neuroimage 237, 118143. 10.1016/j.neuroimage.2021.118143 33991694

[B55] Kashef R. (2022). ECNN: enhanced convolutional neural network for efficient diagnosis of autism spectrum disorder. Cogn. Syst. Res. 71, 41–49. 10.1016/j.cogsys.2021.10.002

[B56] Kassebaum P. (2022). circularGraph. GitHub. Available online at: https://github.com/paul-kassebaum-mathworks/circularGraph

[B57] Kazuhiro K. Werner R. A. Toriumi F. Javadi M. S. Pomper M. G. Solnes L. B.. (2018). Generative adversarial networks for the creation of realistic artificial brain magnetic resonance images. Tomography 4, 159–163. 10.18383/j.tom.2018.00042 30588501 PMC6299742

[B58] Kim J. Calhoun V. D. Shim E. Lee J.-H. (2016). Deep neural network with weight sparsity control and pre-training extracts hierarchical features and enhances classification performance: evidence from whole-brain resting-state functional connectivity patterns of schizophrenia. Neuroimage 124, 127–146. 10.1016/j.neuroimage.2015.05.018 25987366 PMC4644699

[B59] Korda A. Ruef A. Neufang S. Davatzikos C. Borgwardt S. Meisenzahl E.. (2021). Identification of voxel-based texture abnormalities as new biomarkers for schizophrenia and major depressive patients using layer-wise relevance propagation on deep learning decisions. Psychiatry Res. Neuroimaging 313, 111303. 10.1016/j.pscychresns.2021.111303 34034096 PMC9060641

[B60] Kossen T. Subramaniam P. Madai V. I. Hennemuth A. Hildebrand K. Hilbert A.. (2021). Synthesizing anonymized and labeled TOF-MRA patches for brain vessel segmentation using generative adversarial networks. Comput. Biol. Med. 131, 104254. 10.1016/j.compbiomed.2021.104254 33618105

[B61] Kriegeskorte N. (2008). Representational similarity analysis-connecting the branches of systems neuroscience. Front. Syst. Neurosci. 2, 4 10.3389/neuro.06.004.2008 19104670 PMC2605405

[B62] Kuang D. He L. (2014). “Classification on ADHD with deep learning,” in 2014 International Conference on Cloud Computing and Big Data (Wuhan: IEEE), 27–32. 10.1109/CCBD.2014.42

[B63] Kuntzelman K. M. Williams J. M. Lim P. C. Samal A. Rao P. K. Johnson M. R. (2021). Deep-learning-based multivariate pattern analysis (dMVPA): a tutorial and a toolbox. Front. Hum. Neurosci. 15, 638052. 10.3389/fnhum.2021.638052 33737872 PMC7960649

[B64] Laino M. E. Cancian P. Politi L. S. Della Porta M. G. Saba L. Savevski V. (2022). Generative adversarial networks in brain imaging: a narrative review. J. Imaging 8, 83. 10.3390/jimaging8040083 35448210 PMC9028488

[B65] Lan L. You L. Zhang Z. Fan Z. Zhao W. Zeng N.. (2020). Generative adversarial networks and its applications in biomedical informatics. Front. Public Health 8, 164. 10.3389/fpubh.2020.00164 32478029 PMC7235323

[B66] LeCun Y. Bengio Y. Hinton G. (2015). Deep learning. Nature 521, 436–444. 10.1038/nature14539 26017442

[B67] Levakov G. Rosenthal G. Shelef I. Raviv T. R. Avidan G. (2020). From a deep learning model back to the brain—Identifying regional predictors and their relation to aging. Hum. Brain Mapp. 41, 3235–3252. 10.1002/hbm.25011 32320123 PMC7426775

[B68] Li A. Chen S. Quan S. F. Powers L. S. Roveda J. M. (2020a). A deep learning-based algorithm for detection of cortical arousal during sleep. Sleep 43, zsaa120. 10.1093/sleep/zsaa120 32556242 PMC7734480

[B69] Li H. Habes M. Fan Y. (2017). Deep ordinal ranking for multi-category diagnosis of Alzheimer's disease using hippocampal MRI data. arXiv [preprint]. arXiv:1709.01599. 10.48550/arXiv.1709.01599

[B70] Li H. Menegaux A. Schmitz-Koep B. Neubauer A. Bäuerlein F. J. B. Shit S.. (2021). Automated claustrum segmentation in human brain MRI using deep learning. Hum. Brain Mapp. 42, 5862–5872. 10.1002/hbm.25655 34520080 PMC8596988

[B71] Li Q. Yu Z. Wang Y. Zheng H. (2020b). TumorGAN: a multi-modal data augmentation framework for brain tumor segmentation. Sensors 20, 4203. 10.3390/s20154203 32731598 PMC7435374

[B72] Liu M. Zhang J. Adeli E. Shen D. (2017). “Deep multi-task multi-channel learning for joint classification and regression of brain status,” in Medical Image Computing and Computer Assisted Intervention - *MICCAI 2017*, eds M. Descoteaux, L. Maier-Hein, A. Franz, P. Jannin, D. L. Collins, and S. Duchesne (Cham: Springer International Publishing), 3–11. 10.1007/978-3-319-66179-7_1 PMC5942232 29756129

[B73] Liu S. Liu S. Cai W. Che H. Pujol S. Kikinis R.. (2015). Multimodal neuroimaging feature learning for multiclass diagnosis of Alzheimer's disease. IEEE Trans. Biomed. Eng. 62, 1132–1140. 10.1109/TBME.2014.2372011 25423647 PMC4394860

[B74] Liu S. Liu S. Cai W. Pujol S. Kikinis R. Feng D. (2014). “Early diagnosis of Alzheimer's disease with deep learning,” in 2014 IEEE 11th International Symposium on Biomedical Imaging (ISBI) (Beijing: IEEE), 1015–1018. 10.1109/ISBI.2014.6868045

[B75] Liu S. Utriainen D. Chai C. Chen Y. Wang L. Sethi S. K.. (2019). Cerebral microbleed detection using susceptibility weighted imaging and deep learning. Neuroimage 198, 271–282. 10.1016/j.neuroimage.2019.05.046 31121296

[B76] Loh H. W. Ooi C. P. Aydemir E. Tuncer T. Dogan S. Acharya U. R. (2022). Decision support system for major depression detection using spectrogram and convolution neural network with EEG signals. Expert Syst. 39, 1–15. 10.1111/exsy.12773

[B77] Lu D. Popuri K. Ding G. W. Balachandar R. Beg M. F. (2018). Multimodal and multiscale deep neural networks for the early diagnosis of Alzheimer's disease using structural MR and FDG-PET images. Sci. Rep. 8, 5697. 10.1038/s41598-018-22871-z 29632364 PMC5890270

[B78] Mamoshina P. Vieira A. Putin E. Zhavoronkov A. (2016). Applications of deep learning in biomedicine. Mol. Pharmaceut. 13, 1445–1454. 10.1021/acs.molpharmaceut.5b00982 27007977

[B79] Mao Z. Su Y. Xu G. Wang X. Huang Y. Yue W.. (2019). Spatio-temporal deep learning method for ADHD fMRI classification. Inform. Sci. 499, 1–11. 10.1016/j.ins.2019.05.043

[B80] Mojiri Forooshani P. Biparva M. Ntiri E. E. Ramirez J. Boone L. Holmes M. F.. (2022). Deep Bayesian networks for uncertainty estimation and adversarial resistance of white matter hyperintensity segmentation. Hum. Brain Mapp. 43, 2089–2108. 10.1002/hbm.25784 35088930 PMC8996363

[B81] Ngo G. H. Khosla M. Jamison K. Kuceyeski A. Sabuncu M. R. (2022). Predicting individual task contrasts from resting-state functional connectivity using a surface-based convolutional network. Neuroimage 248, 118849. 10.1016/j.neuroimage.2021.118849 34965456 PMC10155599

[B82] Ning K. Duffy B. A. Franklin M. Matloff W. Zhao L. Arzouni N.. (2021). Improving brain age estimates with deep learning leads to identification of novel genetic factors associated with brain aging. Neurobiol. Aging 105, 199–204. 10.1016/j.neurobiolaging.2021.03.014 34098431 PMC9004720

[B83] Niu X. Zhang F. Kounios J. Liang H. (2020). Improved prediction of brain age using multimodal neuroimaging data. Hum. Brain Mapp. 41, 1626–1643. 10.1002/hbm.24899 31837193 PMC7267976

[B84] Olawunmi Olaboopo G. (2021). Improved motor imagery decoding using deep learning techniques (dissertation), Milwaukee, WS: Marquette University. Retrieved from: https://epublications.marquette.edu/dissertations_mu/1086

[B85] Payan A. Montana G. (2015). Predicting Alzheimer's disease: a neuroimaging study with 3D convolutional neural networks. arXiv [preprint]. arXiv:1502.02506. 10.48550/arXiv.1502.02506

[B86] Petersen R. C. Aisen P. S. Beckett L. A. Donohue M. C. Gamst A. C. Harvey D. J.. (2010). Alzheimer's Disease Neuroimaging Initiative (ADNI): clinical characterization. Neurology 74, 201–209. 10.1212/WNL.0b013e3181cb3e25 20042704 PMC2809036

[B87] Plis S. M. Hjelm D. R. Salakhutdinov R. Allen E. A. Bockholt H. J. Long J. D.. (2014). Deep learning for neuroimaging: a validation study. Front. Neurosci. 8, 229. 10.3389/fnins.2014.00229 25191215 PMC4138493

[B88] Qureshi M. N. I. Oh J. Lee B. (2019). 3D-CNN based discrimination of schizophrenia using resting-state fMRI. Artif. Intell. Med. 98, 10–17. 10.1016/j.artmed.2019.06.003 31521248

[B89] Ramzan M. Dawn S. (2021). Fused CNN-LSTM deep learning emotion recognition model using electroencephalography signals. Int. J. Neurosci. 131, 1–11. 10.1080/00207454.2021.1941947 34121598

[B90] Ras G. Xie N. van Gerven M. Doran D. (2020). Explainable deep learning: a field guide for the uninitiated. arXiv [preprint]. arXiv:2004.14545. 10.48550/arXiv.2004.14545

[B91] Ren B. Wu Y. Huang L. Zhang Z. Huang B. Zhang H.. (2022). Deep transfer learning of structural magnetic resonance imaging fused with blood parameters improves brain age prediction. Hum. Brain Mapp. 43, 1640–1656. 10.1002/hbm.25748 34913545 PMC8886664

[B92] Selvaraju R. R. Cogswell M. Das A. Vedantam R. Parikh D. Batra D. (2020). Grad-CAM: visual explanations from deep networks via gradient-based localization. Int. J. Comput. Vis. 128, 336–359. 10.1007/s11263-019-01228-7

[B93] Shao L. Fu C. You Y. Fu D. (2021). Classification of ASD based on fMRI data with deep learning. Cogn. Neurodyn. 15, 961–974. 10.1007/s11571-021-09683-0 34790264 PMC8572240

[B94] Shen L. Shi J. Dong Y. Ying S. Peng Y. Chen L.. (2020). An improved deep polynomial network algorithm for transcranial sonography-based diagnosis of Parkinson's disease. Cogn. Comput. 12, 553–562. 10.1007/s12559-019-09691-7

[B95] Sheynin S. Wolf L. Ben-Zion Z. Sheynin J. Reznik S. Keynan J. N.. (2021). Deep learning model of fMRI connectivity predicts PTSD symptom trajectories in recent trauma survivors. Neuroimage 238, 118242. 10.1016/j.neuroimage.2021.118242 34098066 PMC8350148

[B96] Shi J. Zheng X. Li Y. Zhang Q. Ying S. (2018). Multimodal neuroimaging feature learning with multimodal stacked deep polynomial networks for diagnosis of Alzheimer's disease. IEEE J. Biomed. Health Inform. 22, 173–183. 10.1109/JBHI.2017.2655720 28113353

[B97] Simonyan K. Zisserman A. (2014). Very deep convolutional networks for large-scale image recognition. arXiv [preprint]. arXiv:1409.1556. 10.48550/arXiv.1409.1556

[B98] Solon A. J. Lawhern V. J. Touryan J. McDaniel J. R. Ries A. J. Gordon S. M. (2019). Decoding P300 variability using convolutional neural networks. Front. Hum. Neurosci. 13, 201. 10.3389/fnhum.2019.00201 31258469 PMC6587927

[B99] Spasov S. Passamonti L. Duggento A. Lió P. Toschi N. (2019). A parameter-efficient deep learning approach to predict conversion from mild cognitive impairment to Alzheimer's disease. Neuroimage 189, 276–287. 10.1016/j.neuroimage.2019.01.031 30654174

[B100] Springenberg J. T. Dosovitskiy A. Brox T. Riedmiller M. (2014). Striving for simplicity: the all convolutional net. arXiv [preprint]. arXiv:1412.6806. 10.48550/arXiv.1412.6806

[B101] Sui J. Jiang R. Bustillo J. Calhoun V. (2020). Neuroimaging-based individualized prediction of cognition and behavior for mental disorders and health: methods and promises. Biol. Psychiatry 88, 818–828. 10.1016/j.biopsych.2020.02.016 32336400 PMC7483317

[B102] Suk H.-I. Lee S.-W. Shen D. (2014). Hierarchical feature representation and multimodal fusion with deep learning for AD/MCI diagnosis. Neuroimage 101, 569–582. 10.1016/j.neuroimage.2014.06.077 25042445 PMC4165842

[B103] Suk H.-I. Lee S.-W. Shen D. (2017). Deep ensemble learning of sparse regression models for brain disease diagnosis. Med. Image Anal. 37, 101–113. 10.1016/j.media.2017.01.008 28167394 PMC5808465

[B104] Supekar K. de los Angeles C. Ryali S. Cao K. Ma T. Menon V. (2022). Deep learning identifies robust gender differences in functional brain organization and their dissociable links to clinical symptoms in autism. Br. J. Psychiatry 220, 202–209. 10.1192/bjp.2022.13 35164888 PMC9376194

[B105] Tan C. Sun F. Kong T. Zhang W. Yang C. Liu C. (2018). A survey on deep transfer learning. arXiv preprint arXiv:1808.01974. 10.1007/978-3-030-01424-7_27

[B106] Thanjavur K. Hristopulos D. T. Babul A. Yi K. M. Virji-Babul N. (2021). Deep learning recurrent neural network for concussion classification in adolescents using raw electroencephalography signals: toward a minimal number of sensors. Front. Hum. Neurosci. 15, 734501. 10.3389/fnhum.2021.734501 34899212 PMC8654150

[B107] Thodoroff P. Pineau J. Lim A. (2016). Learning robust features using deep learning for automatic seizure detection. arXiv preprint arXiv:1608.00220. 10.48550/arXiv.1608.0022032172613

[B108] Thompson N. C. Greenewald K. Lee K. Manso G. F. (2020). The computational limits of deep learning. arXiv preprint arXiv:2007.05558. 10.48550/arXiv.2007.05558

[B109] Treacher A. H. Garg P. Davenport E. Godwin R. Proskovec A. Bezerra L. G.. (2021). MEGNet: automatic ICA-based artifact removal for MEG using spatiotemporal convolutional neural networks. Neuroimage 241, 118402. 10.1016/j.neuroimage.2021.118402 34274419 PMC9125748

[B110] Treder M. S. (2020). MVPA-light: a classification and regression toolbox for multi-dimensional data. Front. Neurosci. 14, 289. 10.3389/fnins.2020.00289 32581662 PMC7287158

[B111] Ulloa A. Plis S. Calhoun V. (2018). Improving classification rate of schizophrenia using a multimodal multi-layer perceptron model with structural and functional MR. arXiv preprint arXiv:1804.04591. 10.48550/arXiv.1804.04591

[B112] Ushizima D. Chen Y. Alegro M. Ovando D. Eser R. Lee W.. (2022). Deep learning for Alzheimer's disease: mapping large-scale histological tau protein for neuroimaging biomarker validation. Neuroimage 248, 118790. 10.1016/j.neuroimage.2021.118790 34933123 PMC8983026

[B113] van der Maaten L. Hinton G. (2008). Viualizing data using t-SNE. J. Mach. Learn. Res. 9, 2579–2605.

[B114] Van Essen D. C. Smith S. M. Barch D. M. Behrens T. E. Yacoub E. Ugurbil K. (2013). The WU-Minn human connectome project: an overview. Neuroimage 80, 62–79. 10.1016/j.neuroimage.2013.05.041 23684880 PMC3724347

[B115] Van Hai P. Amaechi S. E. (2021). Convolutional neural network integrated with fuzzy rules for decision making in brain tumor diagnosis. Int. J. Cogn. Inform. Nat. Intell. 15, 1–23. 10.4018/IJCINI.20211001.oa47

[B116] Vieira S. Pinaya W. H. Mechelli A. (2017). Using deep learning to investigate the neuroimaging correlates of psychiatric and neurological disorders: methods and applications. Neurosci. Biobehav. Rev. 74, 58–75. 10.1016/j.neubiorev.2017.01.002 28087243

[B117] Vu H. Kim H.-C. Jung M. Lee J.-H. (2020). fMRI volume classification using a 3D convolutional neural network robust to shifted and scaled neuronal activations. Neuroimage 223, 117328. 10.1016/j.neuroimage.2020.117328 32896633

[B118] Vyas T. Yadav R. Solanki C. Darji R. Desai S. Tanwar S. (2022). Deep learning-based scheme to diagnose Parkinson's disease. Expert Syst. 39, 1–19. 10.1111/exsy.1273928298263

[B119] Wang F. Wu S. Zhang W. Xu Z. Zhang Y. Wu C.. (2020a). Emotion recognition with convolutional neural network and EEG-based EFDMs. Neuropsychologia 146, 107506. 10.1016/j.neuropsychologia.2020.107506 32497532

[B120] Wang L. (2020). Neural network based analysis of resting-state functional magnetic resonance imaging data (dissertation), Riverside, CA: University of California. Retrieved from: https://escholarship.org/uc/item/3sg9r5b0

[B121] Wang S. Shen Y. Chen W. Xiao T. Hu J. (2017). “Automatic recognition of mild cognitive impairment from MRI images using expedited convolutional neural networks,” in ICANN (Alghero, Italy). 10.1007/978-3-319-68600-4_43

[B122] Wang S.-H. Lv Y.-D. Sui Y. Liu S. Wang S.-J. Zhang Y.-D. (2018). Alcoholism detection by data augmentation and convolutional neural network with stochastic pooling. J. Med. Syst. 42, 2. 10.1007/s10916-017-0845-x 29159706

[B123] Wang X. Li X.-H. Cho J. W. Russ B. E. Rajamani N. Omelchenko A.. (2021). U-Net model for brain extraction: trained on humans for transfer to non-human primates. Neuroimage 235, 118001. 10.1016/j.neuroimage.2021.118001 33789137 PMC8529630

[B124] Wang X. Liang X. Jiang Z. Nguchu B. A. Zhou Y. Wang Y.. (2020b). Decoding and mapping task states of the human brain via deep learning. Hum. Brain Mapp. 41, 1505–1519. 10.1002/hbm.24891 31816152 PMC7267978

[B125] Wolterink J. M. Mukhopadhyay A. Leiner T. Vogl T. J. Bucher A. M. Išgum I. (2021). Generative adversarial networks: a primer for radiologists. Radiographics 41, 840–857. 10.1148/rg.2021200151 33891522

[B126] Xia M. Wang J. He Y. (2013). BrainNet viewer: a network visualization tool for human brain connectomics. PLoS ONE 8, e68910. 10.1371/journal.pone.0068910 23861951 PMC3701683

[B127] Xiao G. Shi M. Ye M. Xu B. Chen Z. Ren Q. (2022). 4D attention-based neural network for EEG emotion recognition. Cogn. Neurodyn. 16, 805–818. 10.1007/s11571-021-09751-5 35847538 PMC9279544

[B128] Xu L. Xu M. Ke Y. An X. Liu S. Ming D. (2020). Cross-dataset variability problem in EEG decoding with deep learning. Front. Hum. Neurosci. 14, 103. 10.3389/fnhum.2020.00103 32372929 PMC7188358

[B129] Yan W. Calhoun V. Song M. Cui Y. Yan H. Liu S.. (2019). Discriminating schizophrenia using recurrent neural network applied on time courses of multi-site FMRI data. eBioMedicine 47, 543–552. 10.1016/j.ebiom.2019.08.023 31420302 PMC6796503

[B130] Yan W. Plis S. Calhoun V. D. Liu S. Jiang R. Jiang T.-Z.. (2017). “Discriminating schizophrenia from normal controls using resting state functional network connectivity: a deep neural network and layer-wise relevance propagation method,” in 2017 IEEE 27th International Workshop on Machine Learning for Signal Processing (MLSP) (Tokyo: IEEE), 1–6. 10.1109/MLSP.2017.8168179

[B131] Yan W. Zhao M. Fu Z. Pearlson G. D. Sui J. Calhoun V. D. (2022). Mapping relationships among schizophrenia, bipolar and schizoaffective disorders: a deep classification and clustering framework using fMRI time series. Schizophr. Res. 245, 141–150. 10.1016/j.schres.2021.02.007 33676821 PMC8413409

[B132] Yang D. Hong K.-S. (2021). Quantitative assessment of resting-state for mild cognitive impairment detection: a functional near-infrared spectroscopy and deep learning approach. J. Alzheimers Dis. 80, 647–663. 10.3233/JAD-201163 33579839

[B133] Yang J. Lei D. Qin K. Pinaya W. H. L. Suo X. Li W.. (2021a). Using deep learning to classify pediatric posttraumatic stress disorder at the individual level. BMC Psychiatry 21. 535. 10.1186/s12888-021-03503-9 34711200 PMC8555083

[B134] Yang M. Cao M. Chen Y. Chen Y. Fan G. Li C.. (2021b). Large-scale brain functional network integration for discrimination of autism using a 3-D deep learning model. Front. Hum. Neurosci. 15, 687288. 10.3389/fnhum.2021.687288 34149385 PMC8206477

[B135] Yu T. Zhu H. (2020). Hyper-parameter optimization: a review of algorithms and applications. arXiv preprint arXiv:2003.05689. 10.48550/arXiv.2003.05689

[B136] Zeiler M. D. Fergus R. (2013). Visualizing and understanding convolutional networks. arXiv preprint arXiv:1311.2901. 10.48550/arXiv.1311.2901

[B137] Zeman A. A. Ritchie J. B. Bracci S. Op de Beeck H. (2020). Orthogonal representations of object shape and category in deep convolutional neural networks and human visual cortex. Sci. Rep. 10, 2453. 10.1038/s41598-020-59175-0 32051467 PMC7016009

[B138] Zeng L.-L. Wang H. Hu P. Yang B. Pu W. Shen H.. (2018). Multi-site diagnostic classification of schizophrenia using discriminant deep learning with functional connectivity MRI. eBioMedicine 30, 74–85. 10.1016/j.ebiom.2018.03.017 29622496 PMC5952341

[B139] Zhang J. Feng F. Han T. Gong X. Duan F. (2022a). Detection of autism spectrum disorder using fMRI functional connectivity with feature selection and deep learning. Cogn. Comput. 4, 1–20. 10.1007/s12559-021-09981-z32711819

[B140] Zhang Y. Cai H. Nie L. Xu P. Zhao S. Guan C. (2021). An end-to-end 3D convolutional neural network for decoding attentive mental state. Neural Netw. 144, 129–137. 10.1016/j.neunet.2021.08.019 34492547

[B141] Zhang Y. Lu Q. Monsoor T. Hussain S. A. Qiao J. X. Salamon N.. (2022b). Refining epileptogenic high-frequency oscillations using deep learning: a reverse engineering approach. Brain Commun. 4, fcab267. 10.1093/braincomms/fcab267 35169696 PMC8833577

[B142] Zhao G. (2019). Developing Deep Learning and Bayesian Deep Learning Based Models for MR Neuroimaging. Available online at: https://www.proquest.com/openview/9e379937028054ee808f9c46e5769240/1?pq-origsite=gscholar&cbl=18750&diss=y

[B143] Zhao K. Duka B. Xie H. Oathes D. J. Calhoun V. Zhang Y. (2022). A dynamic graph convolutional neural network framework reveals new insights into connectome dysfunctions in ADHD. Neuroimage 246, 118774. 10.1016/j.neuroimage.2021.118774 34861391 PMC10569447

[B144] Zhou P. Zeng R. Yu L. Feng Y. Chen C. Li F.. (2021). Deep-learning radiomics for discrimination conversion of Alzheimer's disease in patients with mild cognitive impairment: a study based on 18F-FDG PET imaging. Front. Aging Neurosci. 13, 764872. 10.3389/fnagi.2021.764872 34764864 PMC8576572

[B145] Zhou T. Thung K.-H. Zhu X. Shen D. (2019). Effective feature learning and fusion of multimodality data using stage-wise deep neural network for dementia diagnosis. Hum. Brain Mapp. 40, 1001–1016. 10.1002/hbm.24428 30381863 PMC6865441

[B146] Zou L. Zheng J. Miao C. Mckeown M. J. Wang Z. J. (2017). 3D CNN based automatic diagnosis of attention deficit hyperactivity disorder using functional and structural MRI. IEEE Access 5, 23626–23636. 10.1109/ACCESS.2017.2762703

